# Natural Products from the Mediterranean Area as Wound Healing Agents—In Vitro Studies: A Systematic Review

**DOI:** 10.3390/antiox14040484

**Published:** 2025-04-17

**Authors:** Eleftheria Chorti-Tripsa, Vasilis-Zois Galanis, Theodoros C. Constantinides, Christos Kontogiorgis

**Affiliations:** Laboratory of Hygiene and Environmental Protection, Department of Medicine, Democritus University of Thrace, 68100 Alexandroupolis, Greece; echorti@med.duth.gr (E.C.-T.); bill.v.galanis00@gmail.com (V.-Z.G.); tconstan@med.duth.gr (T.C.C.)

**Keywords:** wound healing, in vitro studies, Mediterranean plants

## Abstract

Wound healing is a process that happens when lost tissue replenishes. For this process, both protective elements and wound healing accelerating factors are required. In recent years, the search for natural products that promote faster healing and prevent adverse effects has gained momentum. This is a systematic review, adhering to PRISMA (Preferred Reporting Items for Systematic reviews and Meta-Analyses) criteria, of the wound healing effects of natural products, with a focus on natural products from the Mediterranean region. This study sourced the PubMed and Scopus databases for eligible articles and publications over the last six years. Due to the information volume, only the in vitro studies were included in this review. The criteria set concluded in the 28 studies included. These studies showed that many natural products found in the Mediterranean have been studied for the treatment of wounds. The wound healing effect seems to be related to dose, type of wounded tissue, and application time. Moreover, half of the studies were additionally tested and shown antioxidant activity through DPPH (2,2-diphenyl-1-picrylhydrazyl), ABTS (2,2′-azino-bis(3-ethylbenzothiazoline-6-sulfonic acid), and FRAP (Ferric Reducing Antioxidant Power) assays.

## 1. Introduction

Wound healing is the natural process of tissue repair after injury. It is a physiological phenomenon that is constituted from different phases, and a variety of factors play a pivotal role in this phenomenon. In the first stage, when an injury occurs, in order to prevent excessive bleeding, blood clotting is promoted by cells such as thrombocytes that facilitate this process [1]. Additionally, dead and injured cells are removed from the spot of the injury in addition to any microorganisms present. In this stage, that is called inflammation, cells like neutrophils in association with monocytes accelerate the decontamination of the wound. The following stage is called proliferation and intends to form new tissue. This happens through an array of procedures such as angiogenesis and epithelialization. Endothelial cells migrate to the spot contributing to the form of new blood vessels. Fibroblasts enhance the growth of new tissue and subsequently the re-epithelialization is initiated by the proliferation of epithelial cells. The final stage is called maturation or the remodeling phase and begins when the production levels of collagen equalize to breakdown levels. As the phase advances, the tensile strength, namely the strength of the power that the wound is able to resist, also increases but can never reach the previous primary strength. Moreover, cells that are no longer needed undergo apoptosis. The duration of this phase may take up to 3 months [1].

Wound care originated in ancient times due to a variety of folk remedies from different civilizations. For instance, Egyptians were utilizing bandages crafted with honey to inhibit microbe proliferation [2]. Additionally, plants were pivotal in dealing with wounds. *Salvia officinalis* was used in Turkey to promote wound recovery [3]. The valuable role of *Thymbra capitata* L. in combating wounds in Mediterranean region should also be mentioned [4].

The Ancient Greeks distinguished wounds into two categories, acute and chronic ones. They introduced the concept of washing the wound with boiled water as well as vinegar and wine to cauterize it. Up until the 18th century, which was the onset of surgeries, wound care became medical practice. One century later, antiseptic techniques were introduced as a major discovery and were implemented in medicine. Furthermore, antibiotics contributed to setting a barrier to infections and decreasing the human death rate [2]. Nowadays, even though there are still wound dressings to cure typical wounds, there has been an evolution in the treatment of chronic wounds through the utilization of drugs that soothe pain, such as anti-inflammatory drugs [5].

Reactive oxygen species (ROS) have a special role in wound healing. The concentration of ROS is necessary during the wound healing process to proceed normally; however, ROS must remain within the equilibrium concentration, or it will cause intense inflammation, which leads to anomalous wound closure. This is where antioxidants like vitamin C, vitamin E, several phytochemicals, e.g., curcumin, etc., come into play to maintain the equilibrium of ROS in the wounded area [6,7,8].

In this review, we analyze the wound healing properties of a variety of plants in the Mediterranean region including their chemical constituents and their chemical profile, namely whether they are essential oils or fractions, as well as their total phenolic or flavonoid profile when available. In addition, we evaluate whether the products also have antioxidant activities as well as what percentage of the studies this activity was investigated.

## 2. Methodology

This current systematic review will attempt to implement the PRISMA (Preferred Reporting Items for Systematic reviews and Meta-Analyses) criteria [9] in order to assess the current bibliography on the research that takes place in the Mediterranean region (view Supplementary Materials). This review examines wound healing activity in relation to antioxidant and anti-inflammatory activity as well as the chemical constituents that play a role in these activities of mediterranean natural products.

The data compiled for this review were sourced from Medline (PubMed) and Scopus databases and consist of studies performed using natural products of the Mediterranean area in in vitro wound healing models, within the timeframe 2018–2024. Moreover, the chemical composition of these products was evaluated in parallel with their wound healing activity. The inclusion and exclusion criteria are presented in more detail in Table 1.

**Figure 1:**
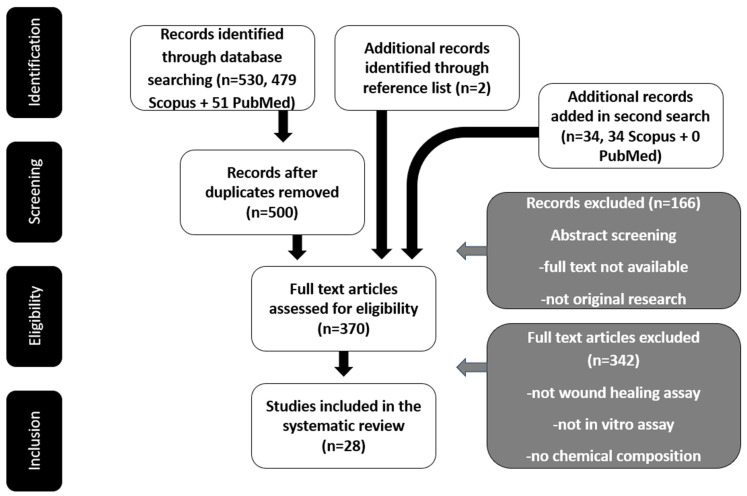
Research flowchart.

The query used in the research engines was (“mediterranean”) AND (“medicinal plant” OR “medicinal herb” OR “aromatic plant”) AND (“wound” OR “wound healing” OR “diabetic foot” OR “antioxidant” OR “anti-inflammatory”). The original filters used were that the studies had to be after 2018 and concern countries around the Mediterranean Sea. This search gathered 566 studies. Of these studies, after applying all the criteria that are presented in detail in Table 1, only 28 were deemed eligible for this research. The whole process is depicted in detail in Figure 1. All three reviewers screened the records, with two conducting the primary and secondary screening and the third cross-examining the resulting records. The natural products tested for their wound healing effects are very diverse and will be analyzed in more detail in the next section.

In order to summarize and better understand the results of this review, the absolute frequency (the number of times a variable has been observed to occur in relation to the total number variables) and relative frequency (1) of certain information, as well as the numerical mean of certain numerical data, which are important in order to evaluate the quality of the studies, were calculated (2).

(1) $$\%Relative\;Frequency=\frac{absolute\;frequency}{total\;number\;of\;values\;for\;the\;variable}\;*\;100$$

(2) $$Numerical\;Mean=\frac{sum\;of\;values}{total\;number\;of\;values}$$

The results of the previous calculations are depicted in pie charts, histograms, and tables.

## 3. Results

### 3.1. Natural Products with Wound Healing Activity: Chemical Composition and Biological Activity

In this review, we present studies that collectively shed light on natural products, which were evaluated as potential anti-inflammatory and antioxidant agents with the ultimate goal to be used in wound healing. The abridged data stated in the following paragraphs can be studied in Table 2 and Table 3.

### 3.2. Calendula arvensis, Lavandula stoechas, and Helichrysum italicum Essential Oils

The waste extracts of the essential oils of *C. arvensis* (C), *L. stoechas* (L), and *H. italicum* (H) were investigated in 2020 by *Addis* et al. in combination (CLH), in pairs *C. arvensis/L. stoechas* (CL) and *C. arvensis/H. italicum* (CH and *L. stoechas/H. italicum* (LH)) and separately (C, L, H). At first, the extracts were tested with the Folin–Ciocalteau method on their total phenolic content with C (384.08 ± 37.58 μg GAE/25 μL), H (663.11 ± 42.71 μg GAE/25 μL), and L (819.62 ± 57.20 μg GAE/25 μL). The main phenolic compounds of each extract were determined by High-performance liquid chromatography (HPLC) as follows: C extract (caffeoylquinic acid derivatives 112.21 ± 6.22 mg/L [10] and Quercetin glycoside derivatives 70.84 ± 5.14 mg/L) [11], H extract (caffeoylquinic acid derivatives 379.76 ± 11.25 mg/L [10] and Naringenin derivatives 24.02 ± 0.87 mg/L), and L extract (caffeoylquinic acid derivatives 77.77 ± 5.20 mg/L, Luteolin derivatives 54.49 ± 5.02 mg/L [12,13,14], Rosmarinic acid 382.46 ± 15.02 mg/L, Rosmarinic acid derivatives 249.80 ± 8.15 mg/L [15], and Apigenin derivatives 18.40 ± 1.55 mg/L) [16].

The waste extracts were also able to exhibit antioxidant properties in ABTS assay in comparison to Trolox. Furthermore, they were capable of stimulating cell viability as exhibited by MTT (3-(4,5-dimethylthiazol-2-yl)-2,5-diphenyltetrazolium bromide) assay in a 72 h period in addition to being able to improve the wound closure (C: 27.2 ± 0.0% in concentration of 1 μL/mL; L: 29.2 ± 0.5% in concentration of 1 μL/mL; and H: 23.6 ± 0.0% in concentration of 5 μL/mL—maximum wound closure compared to control in 72 h in fibroblasts) [16].

### 3.3. Carpobrotus edulis (L.) N.E.Br. Extract

*Carpobrotus edulis* N.E.Br. extract (CAE) was investigated for skin care use and wound healing and regeneration properties. For that purpose, the leaves of the plant were tested regarding their phytochemical properties using reversed-phase (RP) high-performance liquid chromatography (HPLC) coupled to two detection systems: diode-array detection (DAD) and quadrupole time-of-flight (QTOF) mass spectrometry (MS) (RP-LC-DAD-ESI-MS) and in vitro assays. The content of carbohydrates of CAE was high (28.59% ± 0.68%), while the total phenol content and flavonoid content were, respectively, [101.9 ± 6.0] g GAE (Gallic Acid Equivalent)/kg of dry extract (DE) and [545.9 ± 26.0] g rutin equivalents/kg DE. The predominant phytochemicals present in the extract were phenolic acids (55%) with hydroxycinnamic acids being the main component (51.96%), followed by flavonoids (15.74%), tannins (14.82%), and flavonols (15.74%) [17].

In order to investigate the biological activity of CAE assays using keratinocytes and fibroblasts, as well as cell free assays using elastase, collagenase, and hyaluronidase, were performed. CAE had low cytotoxicity at 1000 μg/mL in HaCaT (Spontaneously Transformed Human Keratinocyte) cells (IC_50_ > 1000 μg/mL) and was able to increase the wound healing rate (83% wound closure at 500 μg/mL, relative to 100% induced by allantoin) as well as collagen production in fibroblasts. It also showed strong dose-dependent antioxidant activity and the inhibition of collagenase (>90% at 500 mg/mL) and hyaluronidase (100% at 1000 mg/mL) [17].

### 3.4. Centaurium spicatum (L.) Fritch Extract

Several extracts of *Centaurium spicatum* (L.) Fritch were examined for their biological and chemical characteristics. By means of UHPLC/ESIQqQ-MS/MS (ultra-high performance liquid chromatography coupled to electrospray ionization and mass spectrometer with triple-quadrupole technology), the content of the extracts was revealed. The highest content in known polyphenolic compounds were detected in ethanol/water extracts with the highest concentration of 187.10 mg/kg. The most dominant compound in all extracts but water was rutin (3.32–125.25 mg/kg) followed by caffeic acid (5.95–20.68 mg/kg) and eriodictyol (6.98–9.69 mg/kg). The antioxidant activity of the extracts was tested in cell free in vitro systems with ABTS (2,2′-azino-bis(3-ethylbenzothiazoline-6-sulfonic acid) and FRAP (Ferric Reducing Antioxidant Power) Assay, DPPH (2,2-diphenyl-1-picrylhydrazyl) scavenging assay, and DEMPO (dialkoxyphosphoryl-nitrones 5-diethoxyphosphoryl-5-methyl-1-pyrroline *N*-oxide) spin trap, which showed that the ethanol/water extracts exhibited antioxidant activity. The best antibacterial activity against Gram-negative bacteria, *Salmonela typhimurium*, *Escherichia coli*, and *Enterobacter cloacae*, were the butanol/ethanol extracts while antifungal activity against *A. versicolor*, *Trichoderma viride*, *Penicillium funiculosum*, and *Candida* were effective with both types of extracts. Also, they showed antibiofilm activity [18].

The cytotoxic concentration of the extracts was calculated using MTT assay in HaCaT keratinocyte (IC > 400 μg/mL). After, the ability of the extracts to promote wound healing was assessed via scratch assay in a monolayer of HaCat cells and showed 41.98% and 27.66% cytotoxicity for the ethanol/water 50:50 and 30:70, respectively [18].

### 3.5. Bioactive Films Based on Chitosan and Cynara cardunculus Leaf Extracts

The bioactive films based on chitosan and *Cynara cardunculus* leaf ethanolic extracts (EtPUAE) were tested for cytotoxic effects in Bj5-ta (hTERT-immortalized fibroblast) human skin fibroblasts using MTT with no cytotoxic effects in the maximum concentration of chitosan +5% EtPUAE with 75.6 ± 5.7% cell viability in 6 h and 81.4 ± 8.9% in 24 h. EtPUAE was chemically characterized in a previous publication of the same authors regarding its content in cynaropicrin (1.714 mg/mL), glucose (0.121 mg/mL), fructose (0.071 mg/mL), and total phenolic content (0.149 mg/mL) [19]. The anti-inflammatory effect of the film was examined in Bj5-ta cells via Interleukin 6 (IL6)-mediated inflammation where the strongest effect was noted from the chitosan + 5% EtPUAE film, which reduced IL6 expression by 86% and 83% relative to LPS (bacterial lipopolysaccharide) and plain chitosan film. Finally, the film showed better wound healing activity than the control or 0.177 μg/mL cynaropicrin [20].

### 3.6. Extracts from Ephedra foeminea Forssk Fruits

*Ephedra foeminea* is a plant cultivated predominantly in the Eastern Mediterranean region that is used as a remedy to counter inflammatory diseases. This plant is characterized for its antioxidant, anti-inflammatory, and anti-proliferative actions [21,22].

The main constituent of these species is ephedrines, a type of alkaloid that changes in content among the plant’s species. For the purposes of the experiments, this plant was extracted in four solvents, namely ethanol, hexane, ethyl acetate/water, and methanol/water at a rate of 50:50 [23]. Each fraction underwent HPLC analysis to be checked for substances. The major components identified were thirty-one flavonoid glycosides, which include flavonols (Quercitin, Myricetin, Isorahmnetin, kaempferol), flavones (Apegnin), and anthocyanins (Cyanidin, Malvidin). All extracts contained Quercetin-O-rhamoniside and cyanidin-O-rhamnoside as the main components. In continuation, all fractions were subjected to antioxidant assays, such as the FRAP assay as well as the DPPH and ABTS assays [23].

Moreover, every fraction underwent total phenol and flavonoid analysis. Furthermore, the HECV (human endothelial cell line) cell viability was measured after exposure to the fractions, while the antioxidant potential of the fraction was also investigated using ROS production assay, lipid peroxidation assay, nitrate level calculation, and micronucleus test [23]. Finally, an in vitro wound healing assay was conducted on the HECV cell line, treating cells with H_2_O_2_ under the action of ethyl acetate/water extract. The fraction concentrations applied were 25 μg/mL and 50 μg/mL. Images were taken between 6 and 24 h after the first incision [23].

The results showed that the 50 μg/mL concentration had the greatest ability of wound healing, whose activity managed to close the wound at a rate of 20% after 6 h and 80% after 24 h, while the 25 μg/mL concentration was able to reduce the wound to 77% of the initial wound. It can be deduced that the 50 μg/mL extract promoted wound closure [23]. This could be due to the flavonoid enrichment of the Epoly extract along with the variety of coumaric [24] and ferulic acid derivatives [24] it possesses [23].

### 3.7. Eucalyptus globulus Leaf EO and Extract

*Eucalyptus globulus* is an indigenous plant in Australia and Europe and is utilized to produce paper [25]. For this article, this plant was subjected to distillation.

Apart from essential oil (EO), the remaining water (hydrodistillation residual water, HRW) after the procedure was stored in the fridge. EO underwent gas chromatography–mass spectrometry, which pinpointed 1,8-cineole (72.3%) and α-pinene (9.4%) as the main constituents of the EO. On the other hand, high-performance liquid chromatography was conducted on HRW to examine its constituents, with 1,8-cineole (72.3%) and α-pinene (9.4%) as the majority of the components. Concurrently, a cell viability experiment was performed on four different cell lines, specifically RAW 264.7 (murine macrophages from tumor induced by Abelson murine leukemia virus), B-16V (murine epithelial cells from a B16 melanoma tumor grown in syngeneic C57BL/6 mice), HaCaT, and NIH 3T3 (fibroblasts isolated from a mouse NIH/Swiss embryo) cells. Moreover, a colorimetric assay was conducted on RAW 264.7 cells for the evaluation of nitric oxide (NO) production. Furthermore, an array of experiments was implemented to assess the anti-senescent, depigmenting, and allergic effects [26].

Finally, a scratch assay was conducted on NIH 3T3 cells. The concentrations applied to EO and HRW were 0.16 μg/mL and 0.8 μg/mL, respectively. Images were taken after 24 h. The results showed that there was a similar effect among the groups, namely the control, EO, and HRW. The closure of the wound area was at a rate of 40%. This can be interpreted as those extracts having low wound healing properties in vitro [26].

### 3.8. Fumaria parviflora

*Fumaria parviflora* is a plant cultivated in Egypt and is in danger due to changes happening in the regional soil because of human intervention. This plant demonstrates a powerful biological profile because of its antioxidant and antimicrobial abilities [27].

In the article by Elsaid et al., in order to induce the production of the isoquinoline alkaloids from aerial parts of *F. parviflora*, it is important for the plant to be in the callus stage. Extracts were collected at that stage and dried and isoquinoline alkaloids were isolated. The extracts were then analyzed by UPLC-MS/MS (Ultra-performance liquid chromatography–mass spectrometry) and the presence of isoquinoline alkaloids in eight of the extracts was affirmed. After being tested for cytotoxicity in human fibroblasts (HFs) and confirming low cytotoxicity (IC_50_ > 100 µg/mL), the preparations were tested via scratch assay in HF. Wound healing activity was calculated after 24, 48, and 72 h from the creation of the scratch. It is not clear what concentration was applied [28].

The results showed that among the plants’ alkaloids, sanguinarine had the greatest cell migration rate, reaching 94% after 72 h, while n-Methylstylopine (92.4%), Dihydrosanguinarine (91%), Protopine (90.5%), Dihydrofumariline (88.4%), α-Hydrastine (85.4%), Fumaramine (76.4%), and Microcarpine (75%) exhibited lower ability, and the control group had the lowest one with a 72% migration rate at 72 h. Scientists inferred that the iminium ion group of sanguinarine contributes to strong wound healing properties [28].

### 3.9. Gelidium corneum Extracts

*Gelidium corneum* seaweed was collected and dried at 60 °C before being extracted in ethanol/water, 70:30 at room temperature (20 ± 2 °C) under stirring for 17 h and then concentrated in a rotary evaporator. The dried extract (F1) was then fractionated in a water insoluble fraction (F2), diethyl ether soluble fraction (F3), ethyl acetate soluble fraction (F4), and aqueous fraction (F5), which were all dried. F1 showed the best extraction yield (23.90 ± 1.29%), followed by F5 (4.13 ± 0.44%). The others exhibited lower yields [29]. The constituents of the extracts, as shown in a previous publication of Matias et al., were investigated with NMR and the main components were fatty acids, sterols, terpenes, pigments, and other lipids [30].

The antioxidant capacity of the extracts was also investigated with F4 having the highest total phenolic content (TPC) at 32.92 ± 6.29 mg gallic acid equivalent (GAE)/g extract, with F3 having the greatest reduction in DPPH with EC_50_ = 399.60 μg/mL and the best result in FRAP assay (49.02 ± 5.27 μM FeSO_4_ EQ/g extract). In ORAC (Oxygen Radical Absorbance Capacity) assay, F4 (3060.00 ± 222.20 μmol Trolox equivalents (TE)/g extract), F3 (2916.00 ± 132.80 μmol TE/g extract), and F2 (2868.00 ± 72.29 μmol TE/g extract) had the most antioxidant capacity [29].

In the cytotoxicity assay (MTT) for HaCaT cells, the most cytotoxic fraction was F3, which stopped affecting cell viability at 100 μg/mL, while no fraction induced inflammation by increasing NO production in RAW 264.7 cells in non-cytotoxic concentrations. Moreover, F1 and F2 had photoprotective action in HaCaT cells after Ultraviolet A and B (UVA-B) radiation, with F1 showing 27.52 ± 5.84% less ROS production than the control. The fractions were also tested for their wound healing ability via scratch assay in HaCaT, with F2 and F5 indicating strong wound healing activity [29].

As F5 showed the most potential, it was then formulated for cosmetic use as an emulsion and was tested by a dermatologist on 10 healthy volunteers (18–65 years old) to check acceptability and assess the product’s efficacy. The volunteers had no adverse effects and the area treated with the cream containing F5 had 4.35% less β-carotene oxidation under UVA irradiation than the control, which was plain cream [29].

### 3.10. Helichrysum italicum Hydrolate

*Helichrisum italicum* hydrolate (HH) was tested regarding its potential for the promotion of wound healing using scratch assay and via microscopy immunostaining in human foreskin fibroblasts 1HFF1) and spermatogonial stem cells (SSC) and gene expression analysis with Quantitative reverse transcription polymerase chain reaction (RT-qPCR) (SCC) to investigate several wound healing markers such as collagen I in both HFF1s and SSCs and Sox2, Oct-4, NANOG, p16, Bmi1, and TERT expression in SCCs. Two different concentrations were used with 20% and 30% concentrations of HH [31].

In microscopy immunostaining for both concentrations and both cell lines, there was an increase in collagen I expression compared to the control [31].

HH during gene expression analysis with RT-qPCR in SCC cells promoted Sox2, Oct-4, Bmi1, and NANOG expression and consequently stemness, while it downregulated p16, which promotes senescence and upregulated TERT, of which the downregulation is related to the aging process. As a conclusion, HH might promote wound healing activity [31].

In a previous publication, Serra et al., in 2023, analyzed the consistency of HH using High-performance liquid chromatography with diode-array detection (HPLC-DAD). The two main groups that constitute HH were related to caffeoylquinic acid derivatives (421 ± 20 mg/L) and naringenin derivatives (27 ± 1 mg/L). Moreover, dilutions of 40%, 30%, 20%, and 10% were tested via scratch assay using HFF1 and SSCs and the wound healing rate was measured at 24 h and 48 h. The 30% and 20% concentration scratches closed completely after 48 h while for the untreated cells, the 10% and the 40% concentrations showed reduced migration speed [32].

### 3.11. Juglans regia Leaves

The *Juglans regia* extract’s chemical composition, antioxidant, anti-tyrosinase, antimicrobial, anti-inflammatory, cytotoxicity, and wound healing properties were assessed. The chemical composition of the extract was investigated with the use of HPLC. The compound with the highest concentration was 3-O-Caffeoylquinic acid (18.30 ± 0.04 mg/g)—a known antioxidant [10]—followed by Quercetin 3-O-glucoside (3 6.70 ± 0.19 mg/g), which has shown high antioxidant activity [11], and 3-p-Coumaroylquinic acid (2 5.78 ± 0.30 mg/g), which is a possible antioxidant [24,33]. The antioxidant activity shown by the extract was 137 ± 10 μg/mL for DPPH scavenging activity, 27.6 ± 0.02 μg/mL for reducing power, and 11.83 ± 1.06 μg/mL for Thiobarbituric acid reactive substance (TBARS) formation inhibition. The extract also showed hematoprotective activity, 50% inhibition of tyrosinase at 751 ± 0.01 μg of extract/mL, and antibacterial activity against *P. vulgaris*, *S. lugdunensis*, *S. epidermidis*, and *S. aureus* [34].

In RAW264.7 cell culture, the inhibition of NO production was assessed, with the results showing an 18-fold reduction in NO in comparison to the control. The cytotoxicity of the extract was evaluated with MTT assay in HaCaT cells, and, in continuation, the extract’s wound healing promotion ability was assessed via Scratch assay in the monolayer HaCaT cells with no significant results. However, the addition of the extract in HaCaT cell culture protected the culture from *P. vulgaris* [34].

### 3.12. Lavandula austroapenina (Lamiaceae)

*Lavandula austroapenina* is a plant that thrives in Italy. It has been utilized not only for curative reasons but also for adornment purposes [35].

The article prepared by Gravina et al. analyzes the biological profile of a plant’s five organs, specifically the corolla, calyx, leaf, stem, and root. Firstly, each organ was lyophilized and grinded. Then, each of them were subjected to ultrasound-assisted soaking, using n-hexane and methanol as solvents. After that, alcoholic extracts were analyzed with UHPLC to determine which phytochemicals comprised these extracts, which were mostly phenylpropenoic and phenylpropanoic acid derivatives and flavonoids. Moreover, alcoholic extracts of each plant’s organs were examined for antioxidant activity with ABTS, DPPH, and FRAP assays. Concurrently, the latter extracts were to be evaluated for cytotoxic activity. The HaCaT human keratinocyte cell line was cultivated for the MTT assay in order to evaluate the cell viability of the extracts [36].

Finally, the alcoholic extract of each organ was also checked for wound healing properties. The concentrations applied were 1 μg/mL, 5 μg/mL, and 10 μg/mL. Images were taken at 0, 3, 6, 24, and 27 h after the first incision. The results showed that 1 μg/mL had a significant wound healing effect on each of the four organs, all of which had at least an 80% rate after 24 h, while the calyx had about a 60% rate at 1 μg/mL. After the analysis of the results, it was deduced that the benzene ring of hydroxycimnamic acid derivatives may play a crucial role in wound healing activity due to the repression of an enzyme [36].

### 3.13. Limonium pruinosum (L.) Extracts

*Limonium pruinosum* from Wadi Hof Eastern Desert, Egypt, was used to prepare 1:10 extracts in different solvents (petroleum ether, hexane, butyl acetate, acetone, ethyl acetate, acetaldehyde, ethanol, methanol, diethyl ether, and water). The plant was incubated in the solvent for 3 days, then dried at 50 °C. The dried extract was dissolved in dimethyl sulfoxide (DMSO) [37].

The different extracts, in concentrations of 50 μg/mL, were tested for antibacterial effects against *E. coli* and *S. aureus* (4 × 105 colony forming units (CFU)/mL) using well diffusion assay. The inhibition zone of the ethyl acetate extract (LPEA) for *E. coli* was 20 mm and was 23 mm for *S. aureus* versus the positive control (10 μg/mL Amikacin), which was 27 mm and 24 mm, respectively. Also, the minimum inhibitory concentration of the extract was found to be 25 μg/mL for *E. coli* and 12.5 μg/mL for *S. aureus*, while the biofilm inhibition was 81.05% for E. coli and 75.14% for *S. aureus* [37].

The total phenolic content of the LPEA was determined to be 210 ± 0.11 μg GAE/mg via the Folin–Ciocalteau method. Moreover, the antioxidant activity of LPEA was assessed using the DPPH (IC_50_ = 35.88 ± 2.2 μg/mL), nitric oxide scavenging (IC_50_ = 51.31  ±  1.06 μg/mL), and -OH radical scavenging (IC_50_ = 65.87  ±  1.19 μg/mL) assays. LPEA was analyzed using Gas Chromatography – Mass Spectrometry (GC-MS), with RTX-5 capillary column and helium carrier at 300 °C, and 15 active compounds [Formamide, N, N-dimethyl-, Benzene, (1-butylheptyl)-, Benzene, (1-pentylhexyl)-, Benzene, (1-butylnonyl)-, Trimethyl (4-(1,1,3,3, -tetramethyl butyl) phenoxy) silane, 1,2,4-Triazole-3-amine, 2-Octadecyl-propane-1,3-diol, Decahydro-8a-ethyl-1,1,4a,6-tetramethylnaphthalene, Eicosane, Hexadecane, Cyclohexane, 1,1′-(2-tridecyl-1,3-propanediyl) bis, Tetrapentacontane, 1,54-dibromo, 1,2-Benzisothiazol-3-amine, 1-Docosene, Tetradecanoic acid] were found in the extract, all of which have been reported to have antimicrobial activity and some of which have antioxidant, anti-inflammatory, or anti-cancer activity [37].

LPEA was assessed for its wound healing activity in human gastric epithelial (GES-1) cells via the scratch assay and the wound closure rate was found to be 79.9343  ±  1.98% at 48 h compared to the negative control, which had a wound closure rate of 68.3637  ±  2.32% [37].

Finally, LPEA cytotoxic levels were determined in human diploid fibroblasts (Wi-38), breast cancer cells (MCF-7), and HepG2 cells using MTT. In Wi-38 cells, even at high concentrations (10 mg/mL and 25 mg/mL), there was no apparent cytotoxicity, but in the cancer cell lines, MCF-7 and HepG2, there was 98.9 and 95.8% inhibition, respectively, at 1000 μg/mL, while the IC_50_ was 96.73  ±  2.18 μg/mL for MCF-7 and 81.81  ±  0.99 μg/mL for epithelial-like hepatocellular carcinoma (HepG-2) cells. LPEA was also found to decrease the cell division in the G1 phase in MCF-7 from 58.02 to 55.3% and increase the apoptosis and necrosis at 31.83% and 4.01%, respectively, compared to the control that had a percentage close to 0% [37].

### 3.14. Malva sylvestris Extracts

*Malva sylvestris* (MS) is a plant that thrives in Europe, Asia, and North Africa and is characterized for its anti-inflammatory and antioxidant activity [38]. *Malva sylvestris* extracts are presented to be coated with poly(vinylpyrrolidone)(PVP)/Sodium Alginate (Alg) as potential bandages for wounds [39].

Firstly, PVP and Alg are polymers that can release chemical compounds after entry to the human organism. Flowers and leaves of this plant were prepared by pulverization. Then, the flower dust was combined with water and the leaf dust was blended with ethanol. The following procedure involved centrifuging each of these mixtures. Furthermore, extracts were filtered by nylon filter 0.45 μm for the flower extract and PTFE (Polytetrafluoroethylene) filter 0.45 μm for the leaf extract [39].

A bilayer was formed, with each of two layers, namely the bottom and top, comprising different substances. The bottom layer is made by disintegrating PVP and glycerol in the mixture of the leaf extract and ethanol. For the creation of the top layer, two different PVP/Alg weight ratio solutions were tested. Specifically, the mixture of the flower extract and water was blended with glycerol and with PVP/Alg at two different ratios, 50:50 (*w*/*w*) and 70:30 (*w*/*w*). In continuation, scanning electron microscopy was used to inspect the morphological characteristics of the bilayer films. Then, Ultraviolet and visible radiation (UV-Vis) spectroscopy, Ultraperformance Liquid Chromatography, and Attenuated Total Reflection–Fourier Transform Infrared spectroscopy were used to analyze the extracts. The main active component of the flower extract was anthocyanins and ferulic acid was the main component of the leaf extract [39].

Additionally, in vitro and in vivo wound healing assays were applied to HaCaT cells and mice, respectively. Finally, Enzyme Linked Immunosorbent Assay (ELISA) assay was used to check the expression of IL-6 and Interleukin 1β (IL-1β) cytokines. In respect of the in vitro wound scratch assay, the concentrations applied were 1, 2, and 4 mg/mL of each extract. Images were taken at 24 and 48 h after the first incision. The results demonstrated that MS extract’s bilayer 70:30 ratio at 4 mg/mL had the greatest wound healing properties at 48 h, namely 100%. This is due to the release of two crucial phytochemicals, namely malvidin and ferulic acid, at a higher velocity than the bilayer of 50:50 [39].

### 3.15. Olive Mill Wastewater Extracts

The Olive Mill Wastewater (OMW) was freeze-dried to produce a powder (ELAVF) and dissolved in different solvents (ethanol, acetone, dichloromethane, n-heaxane, ethanol/water 90/10) that displayed high TPC (75.0 mg CT/g), with 34.0 mg CT/g flavonoids and 50.8 mg CT /g phenolic acids. OMW exhibited antioxidant activity with IC_50_ = 0.019 mg/mL against ABTS and IC_50_ = 0.095 mg/mL against DPPH. During HPLC-MS/MS Analysis and NMR, the main constituents of ELAVF were illuminated to be quinic acid [24], caffeic acid [40], and p-Coumaric acid [24] while the second most abundant were Verbascoside residue and Decarboxymethyl-elenolic acid and its derivatives [41].

After the initial screening, PELAVF1S, a pectin polymer containing ELAVF1S, was created. PELAVF1S was then put through NMR and ESI-MS/MS analysis, the results of which were in agreement with the previous ELAVF1S HPLC-MS/MS analysis and NMR. The cytotoxicity of PELAVF1S was evaluated by an NRU test on Balb/3T3 Clone A31 fibroblast cells, which confirmed that PELAVF1S as well as pectin are not cytotoxic. Additionally, the pro-sensitizing potential of the preparation was evaluated by h-CLAT on THP-1 cells, in which PELAVF1S indicated no sensitization and the polymer was also tested in vitro for possibly causing skin irritation using MTT assay on EpiDerm™ RhE inserts, where PELAVF1S showed an increase in cell viability. PELAVF1S, in addition, did not induce hemolysis when tested using peripheral blood and had proliferative effects on BJ fibroblasts and HaCaT cells during MTT assay. Finally, PELAVF1S addition on BJ fibroblasts and HaCaT cells in scratch assay showed an increase in cell motility and faster wound closure, while it was observed microscopically that the introduction of PELAVF1S to HaCaT increases lumican expression and the introduction to BJ fibroblasts increases collagen1 expression, which all indicate promotion of wound healing [41].

### 3.16. Onosma dichroantha Boiss Root Extracts

In 2019, Safavi et al. showed that *Onosma dichroantha Boiss.* cyclohexane extract (CE promotes wound closure. They extracted the plant using cyclohexane (CE), ethyl acetate (EtOAc), and methanol (MeOH) successively and the most potent extract regarding wound healing proved to be CE. The maximum non-cytotoxic concentrations for CE EtOAc and MeOH in RAW264.7 cells were 62.5, 31.25, and 250 μg/mL, respectively, while the maximum non-cytotoxic concentration for CE in HMEC-1 cells was 125 μg/mL. The anti-inflammatory activity of the extracts was investigated by testing their ability to inhibit nitric oxide (NO) production in RAW264.7 cells. CE, EtOAc, and MeOH extracts were able to dose-dependently suppress NO production by 58.5 ± 3.1%, 36.3 ± 4.5%, and 11.0 ± 6.8%, respectively (*p* < 0.05 compared to control). In the fibroblast proliferation assay, only the CE extract showed a 30% increase in Hs27 proliferation at 1000 μg/mL and in scratch assay, it showed a 70% and 92% increase in the wound closure rate in comparison to the control that was 30% and 45% at 16 and 24 h. After the chemical profile of the CE extract was investigated with respect to LC-MS/MS, only three known markers were detected in the extract, shikonin, β, β-dimethylacrylalkannin, and β, β-dimethylacryl shikonin [42].

In continuation to their previous research, in 2023, Safavi et al. decided on fractionation of the CE extract using chromatography and analyzed the separate fractions in order to pinpoint the fraction responsible for the wound healing activity of the extract. The CE extract was made into six fractions (F.A–F.F) with the F.F fraction showing the highest increase in the wound closure rate in human endothelial-like foreskin cells (HMEC-1), 34 and 42% wound closure at 16 and 24 h, respectively, and anti-inflammatory activity (RAW264.7 cells), with dose-dependent decreased production of NO in cells of 45.8 ± 4.3% in comparison to the control, and increasing fibroblast (Hs27) proliferation by 62%. Subsequently, the CE F.F fraction was divided into five sub-fractions. The most active sub-fractions were FF-SUB1 and FF-SUB2 with 51.5 ± 5.2% and 32.9 ± 3.4% inhibition in NO production and 78% and 65% increase in fibroblast growth, respectively. These fractions were, finally, investigated in terms of chemical composition via NMR. The main components identified in FF-SUB1 and FF-SUB2 were Acetylshikonin, β-β-dimethylacrylshikonin, deoxyshikonin, β-hydroxyisovalerylshikonin, and transanethole [43].

### 3.17. Egyptian Opuntia ficus-indica Seed Oils

The Opuntia ficus-indica seed oils (SE (Soxhlet extraction) and UAE (ultrasound-assisted extraction)) phytochemical composition was determined by GC-MS with linoleic acid (SE = 84.99% and UAE = 75.2%) and oleic acid (SE = 8.18% and UAE = 14.01%) being the most abundant fatty acids, with the difference between SE and UAE being the existence of caproic acid and palmitoleic acid only in UAE. In the cytotoxicity assay on normal skin fibroblast cell line (BJ.1), the IC_50_ values were 132.4 and 194.2 μg/mL for SE and UAE, respectively. Finally, both oils promoted wound closure with an SE closure rate = 5% and UAE closure rate = 73% in comparison to the negative control that had a 20% closure rate [44].

### 3.18. Oregano (Origanum vulgare L.) Essential Oil

In 2020, Avola et al. looked into the wound healing and anti-inflammatory activity of *Oreganum vulgare* L. essential oil (OEO), which is known for its antioxidant activity (Walasek-Janusz et al.). Using GC-MS, they were able to recognize the major components of EOE, thymol (25.2 ± 0.27%), p-cymene (21.54 ± 0.35%), and linaool (4.26 ± 0.05%) with monoterpenes as the main chemical family (60.97%). EOE was assessed for its antioxidant activity by DPPH assay and a high SC50 value (114 ± 6 μg/mL) was observed. After, the ROS production decrease was confirmed in epithelial-like cells from human skin (NCTC 2544) cell culture with 2′,7′-dichlorodihydrofluorescein diacetate (H2DCFDA/DCFH-DA/DCFDA), with OEO showing significantly lower ROS levels in comparison to the control, the 8-OHdG generated in NCTC 2544 cells was also reduced in OEO in comparison to the control [45].

Using scratch assay, OEO showed increased cell proliferation and migration rate. Moreover, to test the potential anti-inflammatory effects of OEO, they used RT-PCR to quantify the expression levels of pro-inflammatory protein m-RNA (messenger RiboNucleic Acid) of ICAM-1, iNOS, and COX-2, which showed a decrease with the use of OEO. Lastly, the inhibition of degradation of the extracellular matrix proved to be lower by measuring the expression levels of metalloproteinases MMP-1 and MMP-12 using RT-qPCR [45].

### 3.19. Phlomis Rigida Labill. Extracts

*Phlomis rigida* Labill. is cultivated in Turkey and serves as a wound healing treatment as well as an appetizer in cooking [46,47]. For the experiments, four different fractions were prepared—n-hexane, dichloromethane, ethyl acetate, and methanol [48].

Firstly, the fractions were subjected to analysis for the evaluation of antioxidant activity. Assays were conducted such as ABTS and DPPH as well as Follin–Ciocalteu for the determination of the total phenolic composition of each fraction. The total phenolic content of the extract was 4205 mg of gallic acid equivalent (GAE)/100 g. Additionally, a Lox enzyme inhibition assay was performed to check the fractions’ anti-inflammatory effects. Furthermore, each fraction underwent LC-MS (liquid chromatography–mass spectrometry) and n-hexane was subjected to gas chromatography for further analysis [48].

According to LC-MS, the main flavonoid constituents of the methanolic extracts are luteolin and apigenin, which have wound healing potential [49]. The EO, on the other hand, consists mostly of sesqiterpenoids, with main constituents of β-caryophyllene and β-selinene. The volatile components were analyzed via the n-hexane extract. The most abundant volatile components were hexadecanoic acid (32.0%) [50], neophytadiene isomer (16.8%), with several studies eluding that it has antioxidant and anti-inflammatory activity [50,51,52], myrcene, which has shown antioxidant activity [52,53], pentacosane (8.2%), methyl hexadecanoate (5.7%), and tricosane (4.4%) [48].

Apart from in vitro biological assays, in vivo experiments were also performed. The in vivo wound healing assay was specifically performed on Balb-c mice.

An in vitro wound healing assay was conducted on murine fibroblasts L929 NCT cells, which are mouse embryonic fibroblasts. These cells were exposed to three different concentrations of the plant extract, specifically 0.125, 0.25, and 0.5 mg/mL. As a result, there were four groups, including the control group, as well as those of the three concentrations [48].

Images were captured at 8 and 24 h post-incision. Among the four groups, the results showed that 0.5 mg/mL exhibited the greatest ability of cell migration both at 8 and 24 h, with a percentage of 50.1% and more than 90%, respectively, while 0.25 and 0.125 mg/mL were the second and third, respectively, at both time stamps. Specifically, at 8 h, the 0.125 dose showed 45.72%, while the 0.25 dose had a 49.71% wound closure rate. The control group presented decreased wound healing properties in the two specific hours with respect to the latter three groups, specifically at 42% after 8 h and 90% after 24 h [48].

In a nut shell, it can be deduced that wound closure mediated by this plant extract is dose-dependent, i.e., an increase in the extract’s concentration causes a rise in cell migration [48].

### 3.20. Pistachia vera L. Hull Extract

The genus of *Pistachia* is composed of three species and is cultivated in Middle Eastern and Mediterranean areas. It has been tested clinically and proved effective against diseases such as diarrhea and asthma, as well as promoting wound healing [54,55,56]. In this article, *Pistachia vera* L. underwent extraction with methanol and the obtained extract was then subjected to extraction using the following solvents: CHCl_3_, EtOAc, and n-butanol [57].

Consequently, column chromatography was conducted on the chloroform fraction, then plate chromatography was performed on one sub-fraction, and finally NMR spectroscopy was conducted to identify which compound carries wound healing properties. One compound proved to be 3*α*-hydroxytirucalla-7,24*Z*-dien-26-oic acid, which belongs to triterpenes that are known for their antioxidant and antibacterial activity [58].

Moreover, the extracts were tested on NIH 3T3 cells using cell viability assay. Additionally, RT-qPCR was performed to examine the expression of three factors in the samples, specifically IL-6, TNF-a, and NF-κB1 [57].

Finally, a wound scratch assay was performed on NIH 3T3 fibroblast cells treated with each fraction. Concentrations were in the range of 0.02 to 20 μg/mL, and the fractions of CHCl_3_, EtOAc, and butanol showed the greatest results at 0.02, 0.04, and 0.2 μg/mL, respectively. It is noteworthy that the CHCl_3_ fraction demonstrated the highest cell migration ability with respect to the control at a wound closure rate of 50.63%. Images were taken after 48 h [57].

Further analyses showed that among the five sub-fractions of CHCl_3_, the fourth one presented the highest wound healing ability. Then, that one was divided into three sub-fractions with the aid of preparative thin-layer chromatography. The third one had the greatest wound healing property [57]. One major factor that contributed to the reduction in the wound is the 3α-hydroxymasticadienolic acid that contrives to heal the wound at a higher rate than that of the allantoin action, which is the control group. This terpenoid was identified in the third sub-fraction Fr4 CHCl_3_ fraction at a high concentration, therefore maintaining cell viability and proliferation [57].

Later, in 2023, a follow-up study from Sarkhail et al. focused on the wound healing effects of anacardic acid (13:0), which is a known anti-inflammatory and antioxidant substance [59] isolated from *Pistacia vera* Hull Extract (AA), and is also known for its cytotoxic activity. AA has nonselective cytotoxic activity as the MTT assay showed an IC_50_ = 18.90 μg/mL for MCF-7 (cancer) cells, IC_50_ = 26.10 μg/mL for HepG-2 (cancer) cells, and IC_50_ = 17.73 μg/mL for MKN-45 (human gastric adenocarcinoma) cells, but also IC_50_ = 18.69 μg/mL for NIH 3T3 (normal) cells. Still the cytotoxicity was lower than cisplatin (IC_50_ < 6 μg/mL). Finally, by using scratch assay for NIH 3T3, it was evident that AA had wound healing activity, albeit non-dose-dependent, with wound closure rates of 31.92%, 20.06%, and 29.12%, which are more than the control at 1.25, 2.5, and 5 μg/mL, respectively [60].

### 3.21. Butter Oil (Ghee) Enrichment with Rosemary and Clove Plants

Ghee is a dairy product, mainly produced in India and Middle East countries. Ghee is composed of lipid-soluble vitamins and lipids that are vulnerable to oxidation, thus decreasing its lifespan [61]. This article represents ghee’s butter oil being enriched with rosemary (RG) and clove (CG) plants to analyze the biological profile of ghee and its resistance to oxidation.

Firstly, ghee was purified by melting. It was heated at 110–125 °C, and the bubbles were removed in order to filtrate the remaining liquid (BT—Bovine Traditional). In the purified ghee, the most abundant fatty acid was palmitic acid followed by oleic, myristic, and stearic acids, constituting 81% of the ghee. Later, rosemary and clove plants were added by soaking at a rate of 6% (*w*/*w*) each and were stored in the dark at 60 °C for 3 weeks. Enriched ghee retained more retinol than non-enriched. The assessment of oxidation stability was checked with the peroxide value. The three different samples were subjected to fatty acid analysis and then to HPLC to inspect the volatile compounds. Carnosic acid had the largest concentration in all samples (30 to 77 mg/kg). In the rosemary-enriched ghee, the most plentiful compound was carnosol (8.6 mg/kg) and in the clove-enriched, it was gallic acid (5 mg/kg) [62].

Moreover, normal human fibroblasts (NHF-A12) were cultured and underwent wound scratch assays. The concentration applied was 50 mg/mL. Images were captured at 0, 12, and 24 h after the first incision. It is vital to mention that there were four groups in this assay, namely clove-enriched ghee as well as rosemary-enriched ghee, a positive control (epidermal growth factor - EGF), and a negative control (only fibroblasts). At 12 h, the results showed that CG and RG had identical effects, namely 44.5% and 45.2%, respectively, while the negative control group had the lowest one at a rate of 18.8%, whereas the non-enriched ghee group had a rate of 29.6%. After 24 h, RG and CG had perfectly closed the wound, while the control groups approached the value of 91.5% (positive control group) and 81.8% (negative control group). As a result, it can be inferred that rosemary and clove played a crucial role due to their contribution to wound closure, while ghee itself demonstrated great action too [62]. It should be highlighted that the combination of these plants with ghee, each one separately, had high healing properties due to the addition of vitamin A and E that are encompassed in these two.

### 3.22. Active Metabolites from Salvia officinalis L.

In the article prepared by the author and coworkers, 10 plants from the Lamiaceae family were used for the conduction of experiments that are depicted in Table 1 along with their extracts [63].

Infusions were produced after the distillation of 4 g of the plants’ aerial parts into 200 mL of water, then the filtration and the elimination of the solvent were conducted. Moreover, *Salvia officinalis’*s aqueous extract was subjected to the identification and isolation of its phytochemicals. Furthermore, the cell viability of the 10 plant extracts was determined in NIH 3T3 fibroblasts. Additionally, each extract and infusion underwent a photoprotection assay as well as an intracellular reactive oxygen species assay [63].

Finally, *Salvia officinalis’*s four isolated compounds were subjected to a wound scratch assay. Specifically, salvianic acid A, along with salvianolic acid K, as well as rosmarinic acid [15] and luteolin [12,13,14], underwent scratch assay. The concentrations applied were 0.1 and 1 μg/mL. Images were taken after 24 h. The results showed that salvianolic acid K’s at 1 μg/mL stimulated wound healing at a rate of 70%, while the positive group had a rate of 80%. The lowest percentage of cell migration was demonstrated by the latter phytochemical at a concentration of 0.1 μg/mL [63].

### 3.23. Santolina rosmarinifolia L. Essential Oil

In the article prepared by Alves-Silva, Gonçalves et al., the plant was collected in Portugal, and its essential oil (EO) was assessed for its biological activities and possible antimicrobial effect [64].

Firstly, the plant was air-dried for three consecutive days and protected from light. After that, it was subjected to hydrodistillation for 3 h with s Clevenger-type apparatus. Then, the essential oil was utilized to assess its antifungal activity. The minimum inhibitory concentration (MIC) and minimum lethal concentration (MLC) were measured to evaluate EO’s action against an array of fungal strains. Furthermore, the RAW 264.7 macrophage cell line was cultured for the assessment of anti-inflammatory activity. A NO production assay was conducted along with the analysis of the expression of a variety of genes, namely pro-inflammatory proteins, interleukins, and enzymes [64].

Moreover, a wound healing assay was performed on NIH 3T3 cells, and the concentration applied was 0.58 mg/mL. Images were taken after 18 h of the first incision. The results showed that there was an increased wound healing rate of 91%. It can be inferred that the plant’s EO has notable in vitro wound healing properties [64]; furthermore, it should be noted that chemical compounds that comprise this plant, such as borneol, limonene, and β-pipene, have been found to be associated with high cell migration activity.

### 3.24. Ethanolic Extract of Sarcopoterium spinosum Fruits 

The dried fruits of *Sarcopoterium spinosum* were mixed with ethanol at 50 g/L (SEE). After 3 h, the ethanol extract was collected, filtered, and evaporated at 42 °C in a rotary evaporator. The SEE composition was investigated by HPLC-MS/MS (High-performance liquid chromatography—Mass spectrometry), pinpointing the most abundant group (~50.8%), ellagitannin, and the second and third most abundant, triterpenes (~11.9%) and flavonoids (~7%) [65].

SEE, single PPs, and H_2_O_2_ were also evaluated on HECV cells regarding their cytotoxicity (MTT assay), which showed no cytotoxicity and antioxidant activity (DCFDA TBARS and GSH/GSSG ratio assays). In DCFDA, during H_2_O_2_-insult counteraction reduced fluorescence SEE = −22%, Corilagin (Cg) = −33%, and Quercentin (Qu) = −22% [11] and pre-treatment SEE = −24%, Cg = −24%, and Qu = not significant. In TBARS, during H_2_O_2_-insult counteraction reduced the MDA level SEE = 10.5 ± 2 and Cg = 11.1 ± 1.1, and pre-treatment SEE = −70%, Cg = −72%, and Qu = −64%. In glutathione/ glutathione disulfide (GSH/GSSG) ratio assay during H_2_O_2_-insult counteraction, the GSH/GSSG ratio was increased SEE = −110%, Cg = −109%, and Qu = −111%, and the pre-treatment GSH/GSSG ratio was decreased SEE = 11.3 ± 0.5, Cg = 10.7 ± 1.3, and Qu = 12.3 ± 1.6. All in all, the antioxidant activity of SEE, Cg, and Qu proved better during H_2_O_2_-insult counteraction than pre-treatment [65].

The anti-inflammatory capabilities of SEE, Cg, and Qu were tested via the thermal denaturation of BSA, where Qu had 53% inhibition of the denaturation, SEE had 49%, and the positive control had 60%. Also, the anti-inflammatory activity was investigated for HECV cells while they were insulted with H_2_O_2_ by measuring the NO release. Both counteraction and protection methods of administering were tested and, in general, the counteraction method was deemed more effective by increasing the NO levels by SEE = +82%, Cg = +65%, and = +74% [65].

Finally, the wound healing activity of SEE, Cg, and Qu was checked via the scratch test on HECV cells, while they were insulted with H_2_O_2_ as a counteraction and protection method. While Qu had no significant effect, SEE had a 60% faster wound closure compared to the control and Cg 62% when counteracting, and 64% and 68%, respectively, when protecting [65].

### 3.25. Thymbra spicata L. Extracts

*Thymbra spicata* L. is a plant mostly cultivated in Mediterranean areas. In traditional medicine, it is often used as remedy for diseases such as hyperlipidemia and hyperglycemia [66,67]. Studies have demonstrated that this plant is composed of phenolic acids and flavonoids. For this study, the aerial parts of *Thymbra spicata* L. were extracted into two different solvents: ethanol and water [68].

Firstly, the total phenol and flavonoid content of the extracts was measured. Furthermore, HPLC and GC-MS analyses were conducted in order to identify the phytochemicals that are contained in the extracts. The components with the highest concentrations were monoterpenoic phenols, polyphenolic acids, flavonoids, and their derivatives, with the most abundant being the rosmarinic acid (38.6%) in the aqueous extract with high antioxidant activity [15] and carvacrol in the ethanolic extract (36.84%), which also has high antioxidant activity [69].

Moreover, DPPH assay was performed to analyze the antioxidant activity of the extracts. Additionally, FaO-fat hepatoma cell lines from rats were cultured, ROS production was tested, and triglycerides were calculated in the cells. Also, nitrite levels were determined by spectrophotometric measurement and Western blotting was conducted to examine the percentage of NF-κB p65 protein [68].

Finally, a cell migration assay for the HECV cell line was conducted. The applied concentration of each extract was 1.5 μg/mL and images were taken at 0, 6, and 24 h after the first incision. The results demonstrated that the ethanolic extract had reduced about 80% of the wound size at 6 h and 23% at 24 h, while the aqueous extract was slightly above 80% at 6 h and 32% at 24 h. It can be deduced that the ethanolic extract stimulates cell migration more effectively than the aqueous extract [68]. It should be noted that the aqueous extract was enriched with rosmarinic acid [15] as well as rutin—a potent antioxidant—[70], salvanolic acid, and other phytochemicals, while the ethanolic extract was composed mainly of carvacrol along with thymusin and p-cymene. These chemical compounds might have played a pivotal role in the wound healing process.

### 3.26. Thymus mastichina (L.) L. and Cistus ladanifer L.

Both plants are used for the treatment of wounds and skin disorders. In this specific study, essential oils and hydrolates were produced from each plant’s aerial parts. The essential oils and hydrolates were subjected to GC-MS and GC-FID (Gas Chromatography-Flame Ionization Detector) to check their phytochemical composition, with monoterpene hydrocarbons (71.8%), mainly, α-pinene (50%) and camphene (10.1%), being the main components [71].

Furthermore, each plant underwent a DPPH assay to determine its antioxidant activity. Additionally, an MTT assay was applied to the RAW 264.7 cell culture to monitor cell viability. Moreover, nitric oxide scavenging activity and nitric oxide production was calculated, as well as the minimum inhibitory concentration and the minimum lethal concentration to evaluate the antimicrobial activity of the plants [71].

Finally, a wound healing assay was conducted on the L929 NCTC fibroblast cell line. In addition to the control group, allantoin was used as a positive control for the assay, and its concentration was 1 μg/mL. Images were taken at 12 h. The essential oils’ concentrations were both 0.0002% (*v*/*v*), while the hydrolates’ concentrations were both 2% (*v*/*v*) [71].

*Cistus ladanifer’s* (CL) EO (essential oil) demonstrated excellent cell migration, reaching a percentage of 155.7%, while that of *Thymus mastichina* (TM)’s EO exhibited a rate slightly higher than 100% compared to the positive group. Nevertheless, the TM hydrolate and CL hydrolate stimulated cell migration, namely the TM hydrolate at a rate of 125.1% and the CL hydrolate at a rate of 148.4%. It can be deduced that CL promotes better wound healing than TM [71].

### 3.27. Thymus sipyleus Boiss. Subsp. Rosulans (Borbas) Jalas Extracts

Several extracts of *Thymus sipyleus* Boiss. Subsp. *rosulans* (Borbas) *jalas* exhibited antioxidant activity during the DPPH free radical assay, with the activity ranging between 74.32 and 93.02%. They also showed antibacterial activity against Gram-positive bacteria and, in particular, *S. aureus, S.epidermidis*, and *B. subtilis* [72].

Moreover, the extracts managed to improve the wound closure rate with statistical significance in the scratch assay using fibroblasts and increase the concentration of hydroxyproline in fibroblasts, in general. They also demonstrated anti-inflammatory activity during Griess assay by inhibiting the production of NO in macrophages, with 2.61–50.86% inhibition. The best performing extracts were the infusion (I) and decoction (D) [72].

The HPLC results of I and D showed high luteolin-7-O-glucoside [12,13,14], which was previously considered in in silico research where its derivatives are major targets of transcription factors like Src, NF-κB, MAPK, and SOCS3 [72].

**Table 2:** Collective table of wound healing activity—cell lines and results.

No.	Natural Complex Mixtures	Cell Lines of In Vitro Wound Healing	Wound Closure Rate	Ref.
1	*Calendula arvensis* L. (C), *Helichrysum italicum* (Roth) Don subsp. Microphyllum (Willd.) Nym. (H), *Lavandula stoechas* L. Post-distillation Waste Extracts (L)	HFF1	(-) Control1: 16.2% ± 2.5% 24 h	[16]
			(-) Control1: 20.4% ± 0.4% 48 h	
			(-) Control1: 21% ± 0.0% 72 h	
			C: 21.3% ± 1.68% (1 μL/mL), 20.3% ± 2.9% (5 μL/mL), 20.1% ± 2.5% (10 μL/mL) 24 h	
			C: 26.1% ± 0.8% (1 μL/mL), 22.8% ± 0.4% (5 μL/mL), 23.1% ± 0.6% (10 μL/mL) 48 h	
			C: 27.2% ± 0.0% (1 μL/mL), 23.8% ± 0.0% (5 μL/mL), 24.1% ± 0.0% (10 μL/mL) 72 h	
			L: 21.7% ± 3% (1 μL/mL), 16.7% ± 1.3% (5 μL/mL), 14.1% ± 3.2% (10 μL/mL) 24 h	
			L: 27.4% ± 1.9% (1 μL/mL), 21.7% ± 1.7% (5 μL/mL), 21% ± 3.6% (10 μL/mL) 48 h	
			L: 29.2% ± 0.5% (1 μL/mL), 21.1% ± 0.6% (5 μL/mL), 23.85% ± 0.6% (10 μL/mL) 72 h	
			H: 14.2% ± 3.4% (1 μL/mL), 17.1% ± 3% (5 μL/mL), 14.4% ± 1.9% (10 μL/mL) 24 h	
			H: 19.3% ± 0.2% (1 μL/mL), 22.8% ± 0.3% (5 μL/mL), 16% ± 0.0% (10 μL/mL) 48 h	
			H: 19.9% ± 0.0% (1 μL/mL), 23.6% ± 0.0% (5 μL/mL), 16.4% ± 0.0% (10 μL/mL) 72 h	
			(-) Control2: 24.9% ± 1.8% 24 h	
			(-) Control2: 30.6% ± 2.1% 48 h	
			(-) Control2: 31.8% ± 0.0% 72 h	
			CLH: 27.6% ± 1.6% (1 μL/mL), 21.7% ± 2.7% (5 μL/mL), 19.5% ± 4.2% (10 μL/mL) 24 h	
			CLH: 31% ± 0.0% (1 μL/mL), 29.2% ± 0.9% (5 μL/mL), 27.2% ± 2.5% (10 μL/mL) 48 h	
			CLH: CLOSED (1 μL/mL), 31.2% ± 0.0% (5 μL/mL), 32.3% ± 1.7% (10 μL/mL) 72 h	
			CL: 24.2% ± 3.8% (1 μL/mL), 18.4% ± 3.1% (5 μL/mL), 14.1% ± 1.9% (10 μL/mL) 24 h	
			CL: 29.8% ± 0.8% (1 μL/mL), 27.4% ± 1.9% (5 μL/mL), 22.6% ± 2.2% (10 μL/mL) 48 h	
			CL: 30.4% ± 0.0% (1 μL/mL), 30.1% ± 0.2% (5 μL/mL), 28% ± 1.6% (10 μL/mL) 72 h	
			CH: 23.8% ± 2% (1 μL/mL), 20.8% ± 2.4% (5 μL/mL), 17.6% ± 2.4% (10 μL/mL) 24 h	
			CH: 30.6% ± 0.0% (1 μL/mL), 32.1% ± 0.6% (5 μL/mL), 30% ± 1.5% (10 μL/mL) 48 h	
			CH: CLOSED (1 μL/mL), 33% ± 0.0% (5 μL/mL), 32.6% ± 0.0% (10 μL/mL) 72 h	
			LH: 24.7% ± 1.3% (1 μL/mL), 20.8% ± 2.4% (5 μL/mL), 18.6% ± 1.5% (10 μL/mL) 24 h	
			LH: 30.9% ± 0.7% (1 μL/mL), 28% ± 0.9% (5 μL/mL), 24.2% ± 1.9% (10 μL/mL) 48 h	
			LH: 31.7% ± 0.0% (1 μL/mL), 30.2% ± 0.2% (5 μL/mL), 28.7% ± 1.1% (10 μL/mL) 72 h	
2	*Carpobrotus edulis* L. N.E.Br. Extract (CAE)	HaCaT	(-) Control: ~38%	[17]
			CAE: 83% 24 h (500 μg/mL)	
			(+) Control-Alantoin: 100% 24 h (50 μg/mL)	
3	*Centaurium spicatum* (L.) Fritch extracts	HaCaT	Control: 0.083 ± 0.008% 48 h	[18]
			Cs1: 10.210 ± 1.110% 48 h (400 μg/mL)	
			Cs2: 14.230 ± 1.070% 48 h (400 μg/mL)	
			Cs3: 41.980 ± 2.130% 48 h (400 μg/mL)	
			Cs4: 27.660 ± 1.990% 48 h (400 μg/mL)	
			Cs5: 15.130 ± 2.150% 48 h (400 μg/mL)	
			Cs6: 8.510 ± 0.950% 48 h (400 μg/mL)	
			Cs7: 9.930 ± 0.170% 48 h (400 μg/mL)	
			Cs8: 6.210 ± 0.910% 48 h (400 μg/mL)	
			Cs9: 7.580 ± 1.710% 48 h (400 μg/mL)	
4	*Cynara cardunculus* Ultrasonic Assisted Extraction Pulsed Ethanolic Extract (EtPUAE) in Chitosan Films (CF) (CF + EtPUAE)	Bj5-ta	(-) Control: 13% ± 3% 7 h	[20]
			CF: 17% ± 9% 7 h	
			CF + 1% EtPUAE: 16% ± 6% 7 h	
			(-) Control: 78% ± 6% 24 h	
			CF: 72% ± 8% 24 h	
			CF + 1% EtPUAE: 85% ± 9% 24 h	
5	*Ephedra foeminea* Forssk fruits Ethanol (EE), Methanol/Water (EMW), Hexane (Ehex), Ethyl Acetate/Water (Epoly) extracts	HECV	(-) Control = 66% 24 h	[23]
			Epoly = 75% 24 h (25 μg/mL)	
			Epoly = 77% 24 h (50 μg/mL)	
6	*Eucalyptus globulus* leaf Essential Oil (EO), Hydrodistillation Residual Water Extract (HRW)	NIH 3T3	No significant difference compared to control but slight increase in wound healing	[26]
7	*Fumaria parviflora* Isoquinoline Alkaloids	HF	(-) Control: 47% 24 h, 62.6% 48 h, 72% 72 h	[28]
			9: 67% 24 h, 86% 48 h, 94% 72 h (1 μg/mL)	
			15: 67% 24 h, 83.8% 48 h, 92.4% 72 h (1 μg/mL)	
			12: 64% 24 h, 83% 48 h, 91% 72 h (1 μg/mL)	
			3: 64% 24 h, 82% 48 h, 90.5% 72 h (1 μg/mL)	
			13: 62% 24 h, 78% 48 h, 88.4% 72 h (1 μg/mL)	
			1: 60% 24 h, 71% 48 h, 85.4% 72 h (1 μg/mL)	
			14: 55.6% 24 h, 67.5% 48 h, 76.4% 72 h (1 μg/mL)	
			4: 51% 24 h, 64.4% 48 h, 75% 72 h (1 μg/mL)	
8	*Gelidium corneum* Aqueous/Ethanol Extract Fractions (F1–F5)	HaCaT	(-) Control: 30% 12 h	[29]
			F2: 76.76% ± 10.02% 12 h (600 μg/mL)	
			F5: 61.83% ± 7.25% 12 h (1000 μg/mL)	
			F1, F3, F4: no effect	
9	*Juglans regia* leaf Aqueous/Ethanol Extract	HaCaT	Control: 26.24% ± 2.44% 24 h	[34]
			Extract: 27.86% ± 3.68% 24 h (200 μg/mL)	
10	*Lavandula austroapennina* Alcoholic Extracts	HaCaT	Corolla: 20.55% 6 h (1 μg/mL)	[36]
			Corolla: 100% 24 h (1 μg/mL)	
			Leaf: 33.76% 6 h (1 μg/mL)	
			Leaf: 100% 24 h (1 μg/mL)	
			Stem: 36.79% 6 h (1 μg/mL)	
			Stem: 100% 24 h (1 μg/mL)	
11	*Limonium pruinosum* extract	GES-1	(-) Control: 68.3637% ± 2.32% 48 h	[37]
			EtOAc Extract: 79.9343% ± 1.98% 48 h	
12	*Malva sylvestris* Extracts (Malva)	HaCaT	(-) Control: ~30% 48 h	[39]
			50:50: ~55% 48 h	
			70:30: ~50% 48 h	
			Malva 50:50: ~85% 48 h (1 mg/mL)	
			Malva 50:50: ~85% 48 h (2 mg/mL)	
			Malva 50:50: ~95% 48 h (4 mg/mL)	
			Malva 70:30: ~85% 48 h (1 mg/mL)	
			Malva 70:30: ~98% 48 h (2 mg/mL)	
			Malva 70:30: 100% 48 h (4 mg/mL)	
13	Olive Mill Wastewater Biopolymer Pectin/Ethanolic Extract	BJ, HaCaT	BJ (-) Control: ~23% 24 h	[41]
			BJ Pectin (25 μg/mL): ~35% 24 h	
			BJ Pectin (100 μg/mL): ~35% 24 h	
			BJ PELAVF (25 μg/mL): ~30% 24 h	
			BJ PELAVF (100 μg/mL): ~75% 24 h	
			HaCaT (-) Control: ~47% 24 h	
			HaCaT Pectin (25 μg/mL): ~40% 24 h	
			HaCaT Pectin (100 μg/mL): ~50% 24 h	
			HaCaT PELAVF (25 μg/mL): ~70% 24 h	
			HaCaT PELAVF (100 μg/mL): ~80% 24 h	
14	*Onosma dichroantha* Boiss. Cyclohexane Extract fractions (CE)	HMEC-1	(-) Control: 34% 16 h	[43]
			(-) Control: 42% 24 h	
			FR.F: 76% 16 h (250 μg/mL)	
			FR.F: 76% 24 h (250 μg/mL)	
15	*Onosma dichroantha* Boiss. Root Cyclohexane Extract (CE), Ethyl Acetate (EtOAc), Methanol (MeOH) Extracts	HMEC-1	(-) Control: 30% 16 h	[42]
			(-) Control: 45% 24 h	
			CE: 70% 16 h (125 μg/mL)	
			CE: 92% 24 h (125 μg/mL)	
16	*Opuntia ficus*-indica seed oils Soxhlet extract (SE) and Ultrasound-Assisted Extract (UAE)	BJ.1	(-) Control: ~20% 24 h	[44]
			SE: 73% 24 h (100 μg/mL)	
			UAE: 85% 24 h (100 μg/mL)	
17	*Origanum vulgare* L. Essential Oil (OEO)	NCTC2544	(-) Control: ~25% 48 h	[45]
			OEO: ~63% 48 h (25 μg/mL)	
			(-) Control: ~75% 72 h	
			OEO: ~95% 48 h (25 μg/mL)	
18	*Phlomis rigida* Labill. MeOH Extract	L929 NCTC	(-) Control: 42% 8 h	[48]
			M.E.: 45.72% 8 h (0.125 mg/mL)	
			M.E.: 49.71% 8 h (0.25 mg/mL)	
			M.E.: 50.1% 8 h (0.5 mg/mL)	
19	*Pistacia vera* Anacardic Acid (13:0) From Hull Extract (AA 13:0)	NIH 3T3	(+) Control-Alantoin(50 μg/mL): +33.67% compared to negative control 48 h	[60]
			(AA 13:0): +31.92% compared to negative control 48 h (1.25 μg/mL)	
			(AA 13:0): +20.06% compared to negative control 48 h (2.5 μg/mL)	
			(AA 13:0): +29.12% compared to negative control 48 h (5 μg/mL)	
20	*Pistacia vera* L. Hull MeOH Extracts (Hexane-CHCl3, CHCl3-EtOAc, n-BuOH)	NIH 3T3	(+) Control-Alantoin: 48 h (50 μg/mL)	[57]
			CHCl3: +49.37% more than (+) Control 48 h (0.02 μg/mL)	
			CHCl3 (fr2): +61.6% more than (+) Control 48 h (169 μg/mL)	
			CHCl3 (fr4): +68.2% more than (+) Control 48 h (0.02 μg/mL)	
			CHCl3 (fr4.III): +76.64% more than (+) Control 48 h (0.01 μg/mL)	
			EtOAc: +15.9% more than (+) Control 48 h (0.04 μg/mL)	
			N-BuOH: +34.9% more than (+) Control 48 h (0.2 μg/mL)	
21	*Rosmarinus officinalis* Enriched Ghee (RG)	NHF-A12	(+) Control-EGF(10 nM): 33.23% 12 h	[62]
			Non-enriched ghee: 29.6% 12 h	
			RG: 45.27% 12 h (50 μg/mL)	
			(-) Control: ~21.9% 12 h	
			(+) Control-EGF(10 nM): 91.57% 24 h	
			Non-enriched ghee: 100% 24 h	
			RG: 100% 24 h (50 μg/mL)	
			(-) Control: 81.84% 24 h	
22	*Salvia officinalis* Aqueous Extract	NIH 3T3	(+) Control-FBS 15%: 80% 24 h	[63]
			(-) Control: 37% 24 h	
			Salvianolic Acid K: 70% 24 h (1 μg/mL)	
23	*Santolina rosmarinifolia* Essential Oil (EO)	NIH 3T3	(-) Control: 81% 18 h	[64]
			EO: 91% 18 h (0.58 mg/mL)	
24	*Sarcopoterium spinosum* Fruit Ethanolic Extract (SEE)	HECV	(-) Control: 54% 24 h	[65]
			H2 O2: 25% 24 h (30μM)	
			Counteraction: SEE: 60% 24 h (10 μg/mL)	
			Counteraction: Corilagin (Cg): 62% 24 h (10 μg/mL)	
			Counteraction: Quercentin (Qu): ~30% 24 h (10 μg/mL)	
			Protection: SEE: 64% 24 h (10 μg/mL)	
			Protection: Cg: 68% 24 h (10 μg/mL)	
			Protection: Qu: ~25% 24 h (10 μg/mL)	
25	*Syzygium aromaticum* Enriched Ghee (CG)	NHF-A12	(+) Control-EGF (10 nM): 33.23% 12 h	[62]
			Non-enriched ghee: 29.6% 12 h	
			CG: 44.54% 12 h (50 μg/mL)	
			(-) Control: 21.9% 12 h	
			(+) Control-EGF (10 nM): 91.57% 24 h	
			Non-enriched ghee: 100% 24 h	
			CG: 100% 24 h (50 μg/mL)	
			(-) Control: 81.84% 24 h	
26	*Thymbra spicata* L. Aqueous Extract (T.W.)	FaO, HECV	(-) Control (HECV): 60% 24 h	[68]
			T.W. (HECV): 77% 24 h (1.5 μg/mL)	
	*Thymbra spicata* L. Ethanol Extract (T.E.)		(-) Control (HECV): 60% 24 h	
			T.W. (HECV): 68% 24 h (1.5 μg/mL)	
27	*Thymus mastichina* (L.) L. Essential Oil (TMEO), Hydrolate (TMH) Extracts	L929 NCTC	(+) Control-Alantoin (1 μg/mL): +49.2% than (-) control 12 h	[71]
			TMEO: ~+10% than (-) control 12 h (0.002% v/v)	
			TMH: +25.1% than (-) control 12 h (2% v/v)	
			DMSO (0.002% v/v):~−10% than (-) control 12 h	
	*Cistus ladanifer* L. Essential Oil (CLEO), Hydrolate (CLH) Extracts	L929 NCTC	(+) Control-Alantoin (1 μg/mL): +51.8% than (-) control 12 h	
			CLEO: +55.7% than (-) control 12 h (0.002% v/v)	
			CLH: +48.4% than (-) control 12 h (2% v/v)	
			DMSO (0.002%v/v):~−10% than (-) control 12 h	
28	*Thymus Sipyleus* Boiss. Subsp. Rosulans (Borbas) Jalas Soxhlet Ethanol (SE), Soxhlet n-Hexane (SN), Soxhlet n-Hexane/Ethanol (SNE), Soxhlet Ethanol/n-Hexane (SEN), Maceration Ethanol (ME), Maceration n-Hexane (MN), Maceration n-Hexane/Ethanol (MNE), Maceration Ethanol/n-Hexane (MEN), Decoction (D), and Infusion (I) TS Extracts	NIH 3T3	(+) Control-FGF: 100%	[72]
			(-) Control: 19.4%	
			TS Extracts: 35.74% −82.96% (D~3.6 x (-) Control, I~4.2 x (-) Control)	

**Table 3:** Antioxidant activity—assays and results *.

No.	Natural Complex Mixtures	Antioxidant Assay	Antioxidant Activity Results	Ref.
1	*Calendula arvensis* L. (C) *Helichrysum italicum* (Roth) Don subsp. Microphyllum (Willd.) Nym. (H) *Lavandula stoechas* L. Post-distillation Waste Extract (L)	ABTS	ABTS: LH IC50 = 8.54 ± 0.82 μg/mL	[16]
2	*Carpobrotus edulis* L. N.E.Br. Extract (CAE)	FRAP, ORAC, TEAC, DPPH	FRAP: IC50 = 30.37 μg/mL ORAC: IC50 = 4.41 μg/mL TEAC: IC50 = 36.52 μg/mL DPPH: IC50 = 112.89 μg/mL	[17]
3	*Centaurium spicatum* (L.) Fritch Extracts	FRAP, ABTS, DPPH, -OH Scavencging (DEPMPO/OH)	Cs1: ABTS = 23.46 ± 0.45 mmol GAE/100 mg FRAP = 45.40 ± 1.07 mmol GAE/100 mg DPPH = ~40% DEMPO/OH = ~50%	[18]
			Cs2: ABTS = 37.74 ± 0.74 mmol GAE/100 mg FRAP = 55.13 ± 0.62 mmol GAE/100 mg DPPH = ~43% DEMPO/OH = ~55%	
			Cs3: ABTS = 36.76 ± 0.55 mmol GAE/100 mg FRAP = 53.84 ± 1.29 mmol GAE/100 mg DPPH = ~43% DEMPO/OH = ~55%	
			Cs4: ABTS = 34.47 ± 0.63 mmol GAE/100 mg FRAP = 50.99 ± 0.38 mmol GAE/100 mg DPPH = ~35% DEMPO/OH = ~60%	
			Cs5: ABTS = 21.39 ± 0.23 mmol GAE/100 mg FRAP = 2.70 ± 0.85 mmol GAE/100 mg, DPPH = ~35% DEMPO/OH = ~55%	
			Cs6: ABTS = 29.75 ± 0.55 mmol GAE/100 mg FRAP = 62.41 ± 1.30 mmol GAE/100 mg DPPH = ~35% DEMPO/OH = ~58%	
			Cs7: ABTS = 27.42 ± 0.77 mmol GAE/100 mg FRAP = 54.20 ± 1.34 mmol GAE/100 mg DPPH = ~35% DEMPO/OH = ~55%	
			Cs8: ABTS = 27.18 ± 0.74 mmol GAE/100 mg FRAP = 47.90 ± 1.43 mmol GAE/100 mg DPPH = ~23% DEMPO/OH = ~55%	
			Cs9: ABTS = 24.62 ± 0.69 mmol GAE/100 mg FRAP = 48.51 ± 1.11 mmol GAE/100 mg DPPH = ~25% DEMPO/OH = ~40%	
4	*Cistus ladanifer* L. Essential Oil (CLEO), Hydrolate (CLH) Extracts	DPPH	Poor Antioxidant Capacity	
5	*Ephedra foeminea* Forssk fruits Ethanol (EE), Methanol/Water (EMW), Hexane (Ehex), Ethyl Acetate/Water (Epoly) Extracts	DPPH, ABTS, FRAP, DCFDA (HECV), TBARS (HECV)	DPPH IC_50_: Epoly= 0.99 ± 0.059 mg/mL EMW= 3.2 ± 0.069 mg/mL EE= 4.88 ± 0.11 mg/mL Ehex > 10 mg/mL	[23]
			ABTS IC_50_: Epoly = 0.12 ± 0.03 mg/mL EMW = 0.23 ± 0.005 mg/mL EE = 5.2 ± 0.012 mg/mL Ehex > 10 mg/mL	
			FRAP TEAC: Epoly = 0.37 ± 0.018 mmol TE EMW = 0.15 ± 0.014 mmol TE EE = 0.13 ± 0.01 mmol TE Ehex = N.D. mmol TE	
			DCFDA: +Control (H_2_O_2_) = +44% 30 μM 24 h Epoly = −53% 25 μg/mL & −47% 50 μg/mL 24 h EMW = no effect 24 h EE = −45% 50 μg/mL 24 h	
			TBARS: +control (MDA) = +56% 30 μM Epoly = −39% 25 μg/mL and 50 μg/mL EMW = no effect EE = no effect	
6	*Gelidium corneum* Aqueous/Ethanol Extract Fractions (F1-F5)	DPPH, FRAP, ORAC	DPPH EC50: F1 > 1000 μg/mL F2 = 991.6 μg/mL F3 = 399.6 μg/mL F4 = 973.1 μg/mL F5 > 1000 μg/mL BHT (+Control) = 184.7 μg/mL FRAP: F1 = 31.13 ± 1.31 μM FeSO4/g F2 = 19.22 ± 2.46 μM FeSO4/g F3 = 49.02 ± 5.27 μM FeSO4/g F4 = 19.11 ± 2.54 μM FeSO4/g F5 = 27.96 ± 3.1 μM FeSO4/g BHT (+Control) = 1948 ± 239.1 μM FeSO4/g ORAC: F1 = 46.8 ± 1.17 μmol Trolox/g F2 = 2868 ± 72.29 μmol Trolox/g F3 = 2916 ± 132.8 μmol Trolox/g F4 = 3060 ± 222.2 μmol Trolox/g F5 = 57.58 ± 4.26 μmol Trolox/g BHT (+Control) = 136.4 ± 9.09 μmol Trolox/g	[29]
7	*Juglans regia* leaf Aqueous-Ethanol Extract	DPPH, Ferric Reducing Power (FRAP), OxHLIA, TBARS (HaCaT)	DPPH: EC50 = 137 ± 10 μg/mL RP: EC50 = 27.6 ± 0.02 μg/mL TBARS: EC50 = 11.83 ± 1.06 μg/mL OxHLIA: EC50 (60 min) = 10.8 ± 0.5 μg/mL EC50 (120 mins) = 51 ± 1 μg/mL	[34]
8	*Lavandula austroapennina* Alcoholic Extract	ABTS, DPPH, FRAP	-	[36]
9	*Limonium pruinosum* Extract	DPPH, Hydroxyl Radical Scavenging assay (HRSA)	DPPH: IC50 = 35.88 ± 2.2 μg/mL HRSA: IC50 = 65.87 ± 1.19 μg/mL	[37]
10	*Malva sylvestris* Extracts (Malva)	ABTS	ABTS: RSA(70:30) = 87% RSA(50:50) = 96%	[39]
11	Olive Mill Wastewater Biopolymer Pectin/Ethanolic Extract	DPPH, ABTS	DPPH IC50: LAVF = 0.095 ± 0.003 mg/mL ELAVF1S = 0.152 ± 0.005 mg/mL ELAVF1M = 0.245 ± 0.010 mg/mL ELAVF2S = 0.485 ± 0.021 mg/mL ELAVF2M = 0.599 ± 0.022 mg/mL ELAVF3S = 3.935 ± 0.174 mg/mL ELAVF3M = 1.725 ± 0.074 mg/mL ELAVF4S = 2.052 ± 0.095 mg/mL ELAVF4M = 4.351 ± 0.141 mg/mL ELAVF5S = 0.620 ± 0.017 mg/mL ELAVF5M = 1.028 ± 0.025 mg/mL	[41]
			ABTS IC50: LAVF = 0.0185 ± 0.0007 mg/mL ELAVF1S = 0.0371 ± 0.0012 mg/mL ELAVF1M = 0.0574 ± 0.0021 mg/mL ELAVF2S = 0.1202 ± 0.0043 mg/mL ELAVF2M = 0.1458 ± 0.051 mg/mL ELAVF3S = 2.048 ± 0.0845 mg/mL ELAVF3M = 1.389 ± 0.0428 mg/mL ELAVF4S = 1.789 ± 0.0528 mg/mL ELAVF4M = 3.4862 ± 0.1415 mg/mL ELAVF5S = 0.2581 ± 0.0098 mg/mL ELAVF5M = 0.733 ± 0.0254 mg/mL	
12	*Origanum vulgare* L. Essential Oil (OEO)	DPPH, DCFDA (NCTC2544)	DPPH SC50: OEO = 114 ± 6 μg/mL BHT (+Control) = 8.6 ± 0.3 μg/mL DCFDA: INF-γ/Histamine = 0% reduction in ROS INF-γ/Histamine/Indomethacin: ~74% (10 μM) INF-γ/Histamine/OEO: ~41% (25 μg/mL) Control: ~25%	[45]
13	*Phlomis rigida* Labill. MeOH Extract	DPPH, ABTS, LOX inhibition	DPPH IC50: 0.89 mg/mL ABTS IC50: 0.99 mg/mL LOX inhibition IC50: 19.5 ± 2.8 μg/mL	[48]
14	*Salvia officinalis* Aqueous Extract	DCFDA (NIH 3T3)	DCFDA statistically significant effects: SOW_C (0.01 and 1 μg/mL) SOW_F (1 and 10 μg/mL) SOW_H (0.01 & 1 μg/mL)	[63]
15	*Sarcopoterium spinosum* Fruits Ethanolic Extract (SEE)	DCFDA (HECV), TBARS (HECV), GSH/GSSG ratio (HECV)	Counteraction: DCFDA: H_2_O_2_: +20% (30 μM) SEE: −22% (10 μg/mL) Cg: −33% (10 μg/mL) Qu: −22% (10 μg/mL) TBARS: H_2_O_2_: +97% (30 μM) SEE: −110% (10 μg/mL) Cg: −109% (10 μg/mL) Qu: −111% (10 μg/mL) GSH/GSSG: (-) Control: 9.5 ± 0.3 H_2_O_2_:6.3 ± 0.7 (30 μM) SEE:10.5 ± 2 (10 μg/mL) Cg:11.1 ± 1.1 (10 μg/mL) Qu: no effect (10 μg/mL). Protection: DCFDA: H_2_O_2_:+20% (30 μM) SEE: −24% (10 μg/mL) Cg: −24% (10 μg/mL) Qu: no effect (10 μg/mL) TBARS: H_2_O_2_: +63% (30 μM) SEE: −70% (10 μg/mL) Cg: −72% (10 μg/mL) Qu: −64% (10 μg/mL) GSH/GSSG: (-) Control: 9.5 ± 0.3 H_2_O_2_:6.6 ± 0.6 (30 μM) SEE:11.3 ± 0.5 (10 μg/mL) Cg:10.7 ± 1.3 (10 μg/mL) Qu:12.3 ± 1.6 (10 μg/mL)	[65]
16	*Thymbra spicata* L. Aqueous Extract (T.W.)	DPPH, DCFDA (HECV cells), TBARS (FaO)	DPPH IC50: 25.8 μg/mL ± 1.28 DCFDA 24 h (H_2_O_2_-insulted HECV cells): DCF = −58% (1.5 μg/mL) TBARS MDA level 24 h (+oleate/palmitate): FaO = −70% (0.15 μg/mL), −68% (1.5 μg/mL), −75% (15 μg/mL) HECV = −37% (1.5 μg/mL)	[68]
	*Thymbra spicata* L. Ethanol Extract (T.E.)	DPPH, DCFDA (HECV cells), TBARS (FaO)	DPPH IC50: 24.5 μg/mL ± 0.9 DCFDA 24 h (H_2_O_2_-insulted HECV cells): DCF = −62%(1.5 μg/mL) TBARS MDA level 24 h (+oleate/palmitate): FaO = −110% (0.15 μg/mL), −110% (1.5 μg/mL), −102% (15 μg/mL) HECV = −58% (1.5 μg/mL)	
17	*Thymus mastichina* (L.) L. Essential Oil (TMEO), Hydrolate (TMH) Extracts	DPPH	Poor Antioxidant Capacity	[71]
18	*Thymus sipyleus* Boiss. Subsp. *rosulans* (Borbas) *jalas* Soxhlet Ethanol (SE), Soxhlet n-Hexane (SN), Soxhlet n-Hexane/Ethanol (SNE), Soxhlet Ethanol/n-Hexane (SEN), Maceration Ethanol (ME), Maceration n-Hexane (MN), Maceration n-Hexane/Ethanol (MNE), Maceration Ethanol/n-Hexane (MEN), Decoction (D), and Infusion (I) TS Extracts	DPPH	DPPH IC50: SE = 121.63 ± 1.67 μg/mL, SNE = 100.21 ± 1.19 μg/mL, ME = 104.91 ± 1.04 μg/mL, MNE = 95.19 ± 1.62 μg/mL, D = 43.5 ± 1.02 μg/mL I = 87.38 ± 1.73 μg/mL Ascorbic Acid = 27.63 ± 1.12 μg/mL SN, SEN, MN, MEN = ND	[72]

* The species that are not included in Table 3 have not been tested for their antioxidant activity and no information is given in the provided literature.

## 4. Discussion

This present review used the PRISMA criteria to amass the current new research on the wound healing activity of natural products. This systematic review includes in vitro studies [73] in order to collect and assess the novel knowledge from these studies.

**Figure 2:**
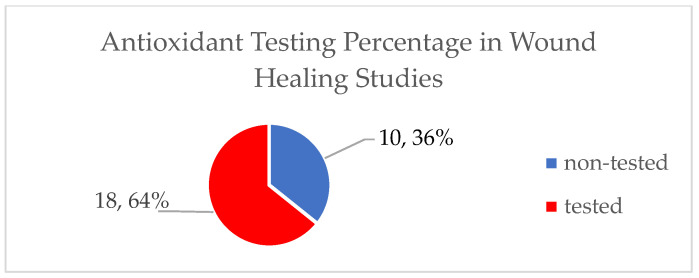
Antioxidant Testing Percentage in Wound Healing Studies.

**Figure 3:**
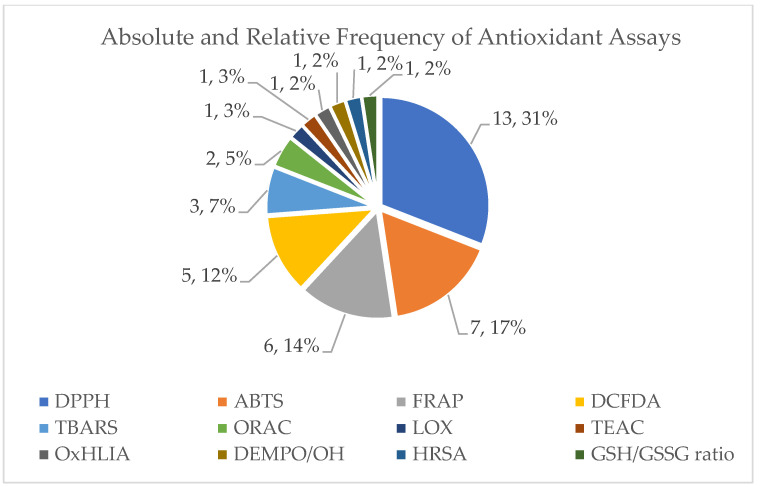
Absolute and relative frequency of antioxidant assays.

All the papers included in this review adhere to certain criteria, as mentioned in Table 1. However, their separate characteristics can be investigated to obtain a better understanding of the types of wound healing in vitro research conducted in the Mediterranean region to obtain some insight into the quality of these studies. Of all 28 articles included in the review, 18 (64%) were also tested for antioxidant activity, while the other 10 (36%) did not include any antioxidant assays (Figure 2). A total of 12 distinct antioxidant assays were used to investigate the antioxidant activity in those 17 articles. The most prominently used assays were DPPH assay (31%), ABTS assay (17%), FRAP assay (14%), and DCFDA assay (12%) (Figure 3).

DPPH, ABTS, and FRAP assays are mostly considered initial screening methods since they do not reflect exactly how the substances, extracts, or EO act when included in a medium or when they enter an organism or cell, as those methods are cell free assays. However, the DCFDA assay gives a pretty good image of the antioxidant activity since it tests that activity on the cell level. As a result, the papers including the DCFDA assay give a more complete image of in vitro testing for antioxidant activity. Moreover, when combined, the in vitro assays can lead to significant results. Finally, in vitro testing for antioxidant activity has the advantage of multiple sample testing in quick sequence [74].

**Figure 4:**
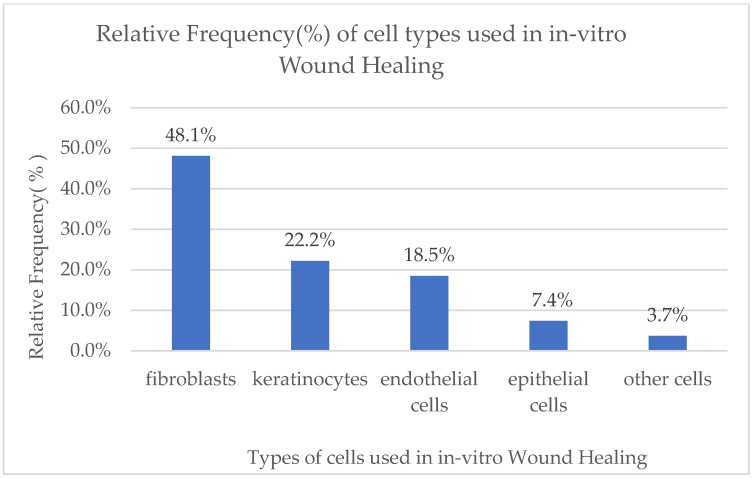
Relative frequency(%) of cell types used in in vitro wound healing.

**Figure 5:**
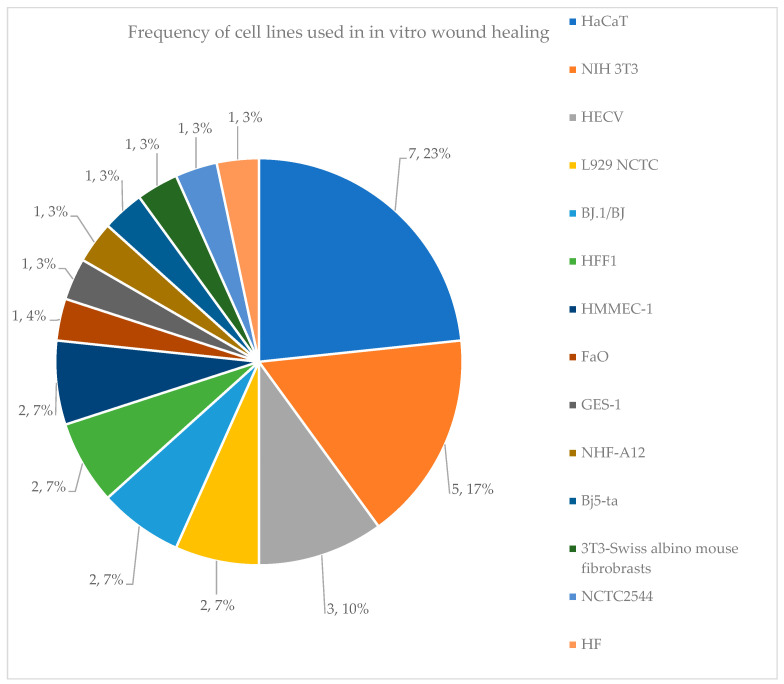
Frequency of cell lines used in in vitro wound healing.

Even though there was no standard cell line used for the in vitro wound healing assays, the cells employed were fibroblasts (48.1%), keratinocytes (22.2%), endothelial (18.5%), epithelial (7.4%), and other (3.7%) cells (Figure 4). In the studied articles, the most prevalently used cell lines were the HaCaT cell line (23%), which are keratinocytes, the NIH 3T3 cell line (17%), which are fibroblasts, and the HECV cell line (10%), which are endothelial cells (Figure 5).

As evident from the previous figures, fibroblasts and keratinocytes are preferred when testing for wound healing activity in vitro since they constitute the key components of the dermis and epidermis [75].

**Figure 6:**
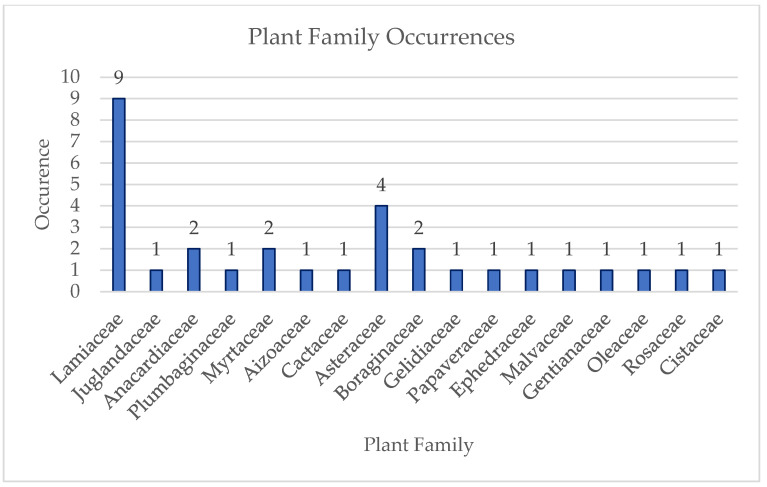
Plant family occurrences.

During the research for this review, it became evident that some plant families were more frequently researched in the Mediterranean region. By far the most frequently encountered plant family is the Lamiaceae family with 11 occurrences, with second most frequently encountered being the Asteraceae family with 4 occurrences. The different plant families in the studied articles as well as their occurrences are depicted in Figure 6.

**Figure 7:**
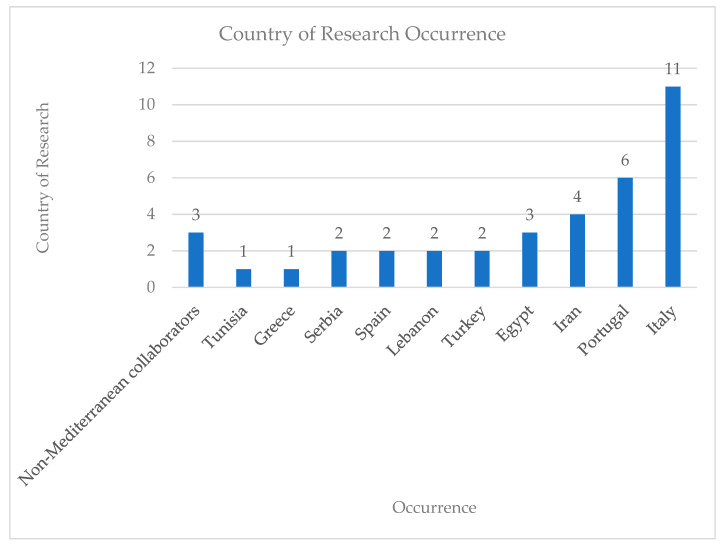
Country of research occurrence.

Moreover, the countries in which the studies came from have an interesting distribution, with Italy taking the lead of publications of in vitro wound healing research in the Mediterranean region with 11 out of 42 country occurrences and Portugal being second with 6 occurrences (Figure 7).

The mean quality of the included articles was investigated by calculating the following metrics: the Mean Number of authors/study = 7.97 ± 2.4, the Mean Impact Factor of the journals where the articles were published (Mean IF = 4.28 ± 1.34), the Mean Number of citations/study = 12.35 ± 15.18, and the Mean Number of Experiments included per study (6.72 ± 2.37) [76].

It is easy to understand why the *Lamiaceae* and *Asteraceae* families are preferred in the Mediterranean region as many species of these families are endemic in this region and are used traditionally for medicinal purposes, while also having antioxidant activity [77].

The presented studies used mainly either fibroblasts or keratinocytes for the wound healing assay (scratch assay). Additionally, the most common family in the Mediterranean was the Lamiaceae family and the most active country in this research is Italy. Additionally, almost half of the studies tested both wound healing and antioxidant activity.

*Calendula arvensis* extracts and essential oils demonstrated great antioxidant activity in several antioxidant assays, such as DPPH free radical scavenging, ABTS, ferric-reducing antioxidant power (FRAP) assay, and β-carotene bleaching assay [78,79,80,81] parallel to showing antibacterial activity against *S. aureus, L. monocytogenes*, and *P. aeruginosa* [78]. In a review written in 2023 by Khouchlaa et al., *C. arvensis* extracts and essential oils demonstrated antimicrobial, antioxidant, and anti-inflammatory properties as well as wound healing activity. The main compounds of the plant essential oils and extracts differ depending on the part of the plant and include several types of compounds such as tannins, alkaloids, polyphenols, flavonoids, and terpenoids [82]. *C. arvensis* also demonstrated antifungal activity [79,83].

Previous research has also exhibited that *Carpobrotus edulis* has strong antioxidant activity [84,85,86,87] and high TPC and TFC [84,85]. Moreover, it has also demonstrated antibacterial activity against *S. aureus* [84,85], *B. cereus* [84,85], and *Enterobacter cloacae* (acetone extract) [86] and weak antibacterial activity against *K. pneumoneae* and *E. coli* [85]. In addition, *C. edulis* is able to protect against oxidative stress and cell death in 158N cells when in 7β-OHC-induced redox status [88]. Lastly, both polar and non-polar extracts of the plant have anti-inflammatory activity, as shown by the 15-lipoxygenase (15-LOX) inhibitory assay and in RAW 264.7 cells by the nitric oxide (NO) inhibition assays using lipopolysaccharide (LPS)-activated assay [89].

*Centaurium spicatum* is not well researched regarding its bioactivity. There have been some studies that suggest antioxidant activity [90], antibacterial and antifungal activity [18], and hepatoprotective activity [91]. However, it is not a well-researched plant.

On the other hand, there are a lot more published studies regarding *Cistus ladanifer* and its biological activities. Most research has concentrated on its strong antioxidant activity [92,93,94,95,96,97,98] and anti-inflammatory activity [97,99]. The plant has also been tested in in vivo models for its wound healing activity [99] and has been researched for cosmeceutical bioactivity [100,101], as well as for use in the industry for packaging applications [102].

*Cynara cardunculus* is another plant that has been relatively well studied as it is an edible plant used in everyday life for cooking and is readily available [103]. Except for its high nutritional value and high phenolic [103,104,105,106] and flavonoid content [105,107], it has demonstrated antioxidant activity [105,106,108,109,110,111,112,113,114,115], anti-inflammatory activity [105,115], antimicrobial properties [110], and laxative activity [109]. However, in this review, the included studies have shown poor antioxidant activity (Table 3).

A plant that is mainly researched regarding its antioxidant activity is *Ephedra foeminea* [23,116,117,118,119]. *Ephedra* spp. has high cathechin, epicatechin, and phenolic contents [116]; however, some studies show that *E. foeminea* does not have high phenolic content [117]. It was also indicated that *E. foeminea* has antihyperglycemic activity [118] and protective effects against oxidative injury [23].

*Fumaria parviflora* has been studied for its antioxidant [120,121,122], hepatoprotective [120,121], and antibacterial activity [122]. So, even though in the included study the samples were not tested (Table 3) for antioxidant activity, there are indications that some antioxidant activity exists. On the other hand, *Gelidium corneum* has been tested for its antioxidant activity in the included study (Table 3) but has also been studied, in addition to its antioxidant [123,124,125] and antibacterial activity [125], for its ability to protect from UV radiation [29,124,126,127] and general dermocosmetic purposes [29,127].

*Helichrysum italicum* has repeatedly shown antioxidant activity [128,129,130,131,132], but has also demonstrated beneficial effects in metabolic syndrome, as shown by the double-blind randomized trial conducted by Kenig et al. in 2022 [133].

*Juglans regia*, also known as walnut, as a common dietary component has special interest in its antioxidant activity [134,135,136,137] but also its anti-inflammatory [134,136], anti-diabetic [137], antibacterial [137], and hepatoprotective [138] activity and has been studied previously for its beneficial effect on healthy and diseased skin [139]. These previously known bioactivities of *J. regia* imply its wound healing activity, as presented in the included study (Table 2).

*Lavandula austroapennina* essential oils, in current research, especially the rich in linalool and terpinen-4-ol, have inhibited the denaturation of BSA (bovine serum albumin) due to heat and the production of nitric oxide in LPS-stimulated macrophages (RAW264.7) [140,141]. Moreover, the lipophilic extracts of the plant have shown antiviral activity in Vero CCL-81 cells infected with herpes simplex virus type 1 (HSV-1), alpha human coronavirus 229E (HCoV-229E), and poliovirus type 1 (PV-1), with most the probable target being the viral envelope [142].

Another well-studied plant is *Lavandula stoechas*, which has known antioxidant [143,144,145,146], anticholinergic [145], hepatoprotective [147], and antibacterial activity (against *Pseudomonas aeruginosa*, *Klebsiella pneumoniae*, *Escherichia coli*, *Enterococcus faecalis*, *Staphylococcus aureus*, *Bacillus cereus*, *Bacillus subtilis*, *Bacillus pumilus*, *Staphylococcus epidermidis*, *Micrococcus luteus*, *Bordetella bronchiseptica*) [143,144,145]. Moreover, *L. stoechas* has also exhibited neuroprotective activity [148] and can provide alleviation from acute lung injury that has been induced by cigarette smoke by modulating the molecular pathways of oxidative stress and NF-κB [149].

In 2014, Boudermine et al. determined in vitro the antioxidant activity of the phenolic compounds of *Limonium pruinosum*, with gallic acid, methyl gallate, and trans-N-caffeoyltyramine being the compounds the highest DPPH scavenging activity [150]. In 2016, Kenouche et al. identified two flavonols from the ethyl acetate extract of *Limonium pruinosum—*3′,7dimethoxyquercetin and 3′methoxyquercetin—that also showed high DPPH scavenging activity [151]. Finally, the antioxidant activity of *L. pruinosum* has been correlated to polyphenol concentration [152].

*Malva sylvestris* has demonstrated its antioxidant activity, as it has a high phenol and flavonoid content and anti-cancer, antimicrobial, and anti-inflammatory activity [153]. Olive oil is likewise known for its antioxidant activity due to its high phenolic content [154,155]. It also has mild antibacterial activity against *P. aeruginosai*, and via gene network analysis and molecular modeling studies, it has shown good bioavailability and stability [155].

There are not a lot of studies on *Onosma dichroantha*; however, the plant has shown antibacterial activity against *E. coli* and *S. aureus* and according to the included studies in this review, it also exhibits promotion of wound healing [156].

*Opuntia ficus* is a widely distributed plant across the Mediterranean basin even though it is not native to the region [157]. Due to its high phenolic content [157,158], it also has strong antioxidant [157,159] and anti-inflammatory activity [158,159]. It has shown potential in the prevention of diabetes complications as well as immunomodulatory activity [157,159].

The most abundant components of *Oregano vulgare* essential oil (OEO) are carvacrol and thymol. Mostly due to these components, OEO exhibits antioxidant, antibacterial, and insecticidal activity [160,161,162]. Likewise, *Phlomis rigida* Labill. EO has demonstrated antibacterial activity [163], while its extract has exhibited anti-inflammatory, analgesic, and wound healing activity both in vitro and in vivo [48]. Moreover, *Pistacia vera* has also shown antioxidant activity as well as antimicrobial, anti-inflammatory, and anti-cancer potential [164,165,166,167].

*Rosmarinus officinalis*, except for antioxidant and antimicrobial activity [168], has also demonstrated anti-cancer activity, as shown in the 2025 study by Darra et al. [169], but can also improve cell viability in Alzheimer’s disease, as shown in the 2025 study by Zhao et al. [170], something that strongly suggests the pro-proliferating role of *R. officinalis* and coincides with the results of the included studies in this review study (Table 2).

*Salvia officinalis* has antioxidant and antimicrobial activity. Bendaas et al. have indicated in their publication in 2025 via molecular docking studies that the antimicrobial activity is probably a result of Type IV topoisomerase, Dihydropteroate synthase, DNA gyrase subunit B, Dihydrofolate reductase, and Penicillin-binding protein 1a inhibition, while the antioxidant activity could be attributed to the inhibition of lipoxygenase and xanthine oxidase [171].

*Santolina rosmarinifolia* extracts and EO contain high phenolic and flavonoid content and exhibit antioxidant activity [172,173]. Similarly, *Sarcopoterium spinosum* methanolic extracts have high total phenolic content (TPC), which has been proved to have strong linear correlation to the antioxidant activity [174]. Likewise, the ethanol extract of *Sarcopoterium spinosum* has high TPC and antioxidant activity as well as bacteriostatic activity [175].

Another common plant with high antioxidant activity is clover, also known as Syzygium aromaticum [176]. The wound healing activity of the ghee infused with S. aromaticum is considerable, according to Table 2. This coincides with other publications that show the prevention of liver injury in rats by pre-treatment with S. aromaticum [177].

*Thymus* spp. has high antioxidant activity since the main constituents of its products are carvacrol and thymol [178] as well as anti-cancer activity [179]. So, it is expected that *T. mastichina* [180,181] and *T. sipyleus* have high antioxidant activity. Additionally, *T. mastichina* exhibit skin repairing and anti-inflammatory activity (Table 2 and Table 3) and *T. sipyleus* demonstrates in addition to antioxidant activity, anticholinergic, antibacterial, and anti-inflammatory activity [71,145,182]. Lastly, *Thymbra spicata* also has high antioxidant activity [183,184], but when using nanoparticles, *T. spicata* extracts have shown promising results in leukemia therapy [185] and the EO and extracts of *T. spicata* have anti-diabetic activity [186].

This study had some initial limitations, such as the articles included were only in English [187] and only accessible by the authors’. The main problems were the large number of initial articles from the initial search and the nonexistence of a standard in the measurement and the control of wound healing or in the cell lines used for that purpose. Moreover, inhomogeneous expression of the results in even the same assays is a limitation. It was also limited to two databases, PubMed and Scopus, and only in vitro studies.

Even with all the limitations, this review showcases that, as is evident in the rest of the prior art, antioxidant activity is related to wound healing [6,188]. However, antioxidant activity assessment is often not included in the screening of new wound healing natural products, as shown previously. Components such as flavonoids and phenols show both strong antioxidant capacity and wound healing activity [6,188]. The regulation of ROS in the wound area is important, in general, in order to contain the inflammation and improve the wound closure procedure [6,7,8]; however, it is even more important to regulate ROS in a diabetic wound since oxidative stress can easily impair the wound healing [189].

Finally, while all articles included showcase wound healing activity, it is not possible to compare their results as the cell lines used as well as the measurement methods differ between each study. Future researchers should take into consideration when starting their research in this field that the protocols of procuring and testing medicinal plant samples and the presentation of the results should be uniform between papers [77].

## 5. Conclusions

This review provided the beneficial effects of natural products derived from the Mediterranean area evaluated as would healing agents. The *Lamiaceae* and *Asteraciae* families were found to present the most significant wound healing and antioxidant activity in the Mediterranean region. Flavonoids, phenols, and terpenoids seem to be the most active components in the case of wound healing activity.

This study enlightened the significance of having standardized ways of measuring in vitro wound healing to make the comparison of the activities of different natural products easier. Even though our systematic review of in vitro research does not directly answer clinical problems, it has a plethora of information that can be used for further analysis in the investigation of potential wound healing agents.

## Figures and Tables

**Table 1:** The inclusion/exclusion criteria of the studies included in the manuscript.

Criterion	Inclusion	Exclusion
Study Design	In vitro studies	Case–control studies Case studies Commentaries Editorials Letters News Notes Guidelines Conference abstracts Animal models Clinical trials
Interventions	Extracts (i.e., from plants, herbs) Essential oils	Pure compounds (i.e., rutin) Non-Natural products Pharmaceutical agents Non-Mediterranean products
Outcomes	Increase in in vitro wound healing rate Chemical composition of natural product	No increase in in vitro wound healing rate No chemical composition included
Language	English	Non-English full text available
Timeframe	January 2018–December 2024	Earlier than January 2018

## Supplementary Materials

The following supporting information can be downloaded at: https://www.mdpi.com/article/10.3390/antiox14040484/s1. Reference [190] is cited in the supplementary materials.

## Glossary

FRAP: ferric reducing antioxidant power; ORAC: oxygen radical absorbance capacity; TEAC: Trolox equivalent antioxidant capacity; DPPH: 2,2-diphenyl-1-picrylhydrazyl assay; ABTS: 2,2′-azino-bis(3-ethylbenzothiazoline-6-sulfonic acid; FRAP: ferric ion reducing antioxidant power; LPS: lipopolysaccharide; ΕΟ: essential oil; TPC: total phenolic content; TFC: total flavonoid content; GAE: gallic acid equivalent; RP-HPLC-DAD-ESI-QTOF-MS: reversed-phase (RP) high-performance liquid chromatography (HPLC) coupled to two detection systems: diode-array detection (DAD) and quadrupole time-of-flight (QTOF) mass spectrometry (MS).
